# European Society of Contact Dermatitis Guideline for Diagnostic Patch Testing—Recommendations on Best Practice (Update 2026)

**DOI:** 10.1111/cod.70195

**Published:** 2026-06-21

**Authors:** Wolfgang Uter, Olivier Aerts, Tove Agner, Richard Brans, Magnus Bruze, Alicia Cannavó, Marie‐Noёlle Crépy, Ana M. Giménez‐Arnau, Margarida Gonçalo, An Goossens, Swen M. John, Carola Lidén, Suzana Ljubojević Hadžavdić, Vera Mahler, Mihaly Matura, Esen Özkaya, Maria Pesonen, Nadia Raison‐Peyron, Thomas Rustemeyer, Radoslaw Spiewak, Luca Stingeni, Ian R. White, S. Mark Wilkinson, Jeanne Duus Johansen

**Affiliations:** ^1^ Department of Medical Informatics, Biometry and Epidemiology Friedrich‐Alexander Universität Erlangen/Nürnberg Erlangen Germany; ^2^ University Hospital Antwerp (UZA) and University of Antwerp Antwerp Belgium; ^3^ Department of Dermatology, Bispebjerg Hospital University of Copenhagen Copenhagen Denmark; ^4^ Institute for Interdisciplinary Dermatologic Prevention and Rehabilitation (iDerm) Osnabrück University Osnabrück Germany; ^5^ Department of Dermatology, Environmental Medicine and Health Theory Osnabrück University Osnabrück Germany; ^6^ Department of Occupational and Environmental Dermatology, Lund University Skåne University Hospital Malmö Sweden; ^7^ Contact Dermatitis and Occupational Dermatoses, Hospital de Clínicas “José de San Martín” Buenos Aires University Buenos Aires Argentina; ^8^ Department of Occupational and Envionmental Diseases, University Hospital of Centre of Paris, Hotel‐Dieu Hospital, AP‐HP, and Department of Dermatology University Hospital of Centre of Paris, Cochin Hospital Paris France; ^9^ Department of Dermatology, Hospital del Mar Research Institute Universitat Pompeu Fabra Barcelona Spain; ^10^ Clinic of Dermatology, University Hospital, Coimbra Local Health Unit and Faculty of Medicine University of Coimbra Coimbra Portugal; ^11^ Faculty of Medicine Catholic University of Leuven (KU Leuven) Leuven Belgium; ^12^ Institute of Environmental Medicine Karolinska Institutet Stockholm Sweden; ^13^ Department of Dermatology and Venereology, University of Zagreb School of Medicine University Hospital Center Zagreb Zagreb Croatia; ^14^ Allergology Division, Paul‐Ehrlich‐Institut Langen Germany; ^15^ Department of Dermatology at Norrlands University Hospital Umeå Umeå Sweden; ^16^ Department of Dermatology at University Hospital of Örebro Örebro Sweden; ^17^ Department of Dermatology and Venereology, Istanbul Faculty of Medicine Istanbul University Istanbul Turkey; ^18^ Finnish Institute of Occupational Health, Occupational Medicine Helsinki Finland; ^19^ Department of Dermatology Montpellier University Hospital and Montpellier University Montpellier France; ^20^ Department of Dermatology‐Allergology and Occupational Dermatology Amsterdam University Medical Centers Amsterdam the Netherlands; ^21^ Department of Experimental Dermatology and Cosmetology Jagiellonian University Medical College, Krakow, Poland and Instytut Dermatologii Krakow Poland; ^22^ Section of Dermatology, Department of Medicine and Surgery University of Perugia Perugia Italy; ^23^ Department of Cutaneous Allergy St. John's Institute of Dermatology, Guy's Hospital London UK; ^24^ Leeds Teaching Hospitals NHS Trust Leeds UK; ^25^ National Allergy Research Centre, Department of Skin and Allergy University of Copenhagen, Gentofte Hospital Copenhagen Denmark

**Keywords:** contact allergy, guideline, patch testing, review

## Abstract

The present guideline updates the initial ESCD patch testing guideline, summarizing all aspects of patch testing for the diagnosis of contact allergy in patients suspected of suffering, or having been suffering, from allergic contact dermatitis or other delayed‐type hypersensitivity skin and mucosal conditions. Sections with brief descriptions and discussions of different pertinent topics are followed by highlighted short practical recommendations, which have been consensualized in a Delphi process. Topics comprise, after an introduction with important definitions, materials, technique, modifications of epicutaneous testing, individual factors influencing the patch test outcome or necessitating special considerations, children, patients with occupational contact dermatitis and drug eruptions as special groups, patch testing of materials brought in by the patient, adverse effects of patch testing and the final evaluation and patient counselling based on this judgement. Finally, short reference is made to aspects of (continuing) medical education and to electronic collection of data for epidemiological surveillance.

**Abbreviations**
ACD: allergic contact dermatitis
CADR: cutaneous adverse drug reaction(s)
CAS (Nr.): chemical abstracts service (numerical substance identifier)
D: day(s) after start of patch test (at D0), for example, D2 is a reading 2 days later
EBS: European baseline series
ESCD: European Society of Contact Dermatitis
ICD: irritant contact dermatitis
INCI: International Nomenclature of Cosmetic Ingredients
INN: International non‐proprietary names (of drugs)
Pet.: petrolatum when used as vehicle for allergen preparations
ROAT: repeated open application test
SDS: safety data sheet (according to CLP/REACH and UN‐GHS)
SmPC: summaries of product characteristics (of medicines)

## 1 Overall Objective and Methods

This updated guideline, based on the initial version published 2015 [1], is intended for dermatologists and other physicians involved in patch testing to identify the responsible (and incidental) contact allergen(s) in patients (including children) in whom allergic contact dermatitis (ACD) or other types of delayed hypersensitivity reaction are suspected, or in whom contact allergy shall rather be excluded. The guideline includes brief information on patch testing materials, techniques, test series, readings, final evaluation, individual or exogenous factors that may influence the outcome of the tests, potential side‐effects and information for patients. Skin testing for immediate‐type hypersensitivity, which is important for the diagnosis of other types of contact reaction, is not dealt with in detail in this guideline. For a more detailed presentation of the patch testing technique and related aspects, the reader is referred to current textbooks [2, 3, 4, 5] and, particularly for aspects of occupational contact dermatitis, to ‘Kanerva's Occupational Dermatology’ [6]. Aspects relating to patch testing children and adolescents, in particular the scope of allergens, have been addressed in recent publications [7, 8].

In September 2024, the European Society of Contact Dermatitis formed a working party to elaborate an up‐date of the 2015 patch test guideline [1]. A call for contributors experienced in the field was made within the Society, including previous authors, but also explicitly approaching younger researchers and clinicians. The resulting international group (see author list) collaborated in the compilation of current information on best practice in diagnostic patch testing, in terms of (i) scrutinizing the first version and (ii) searching for evidence published since 2015. The topics originally defined were still considered valid, and the resulting sections were associated with two to four authors. A pre‐final agreement on the resulting preliminary draft was achieved following two meetings. The resulting recommendations were then put to an online‐voting system (i) concerning agreement with the initial phrasing and (ii) comments for improvement. As several amendments were suggested in this round and then implemented, a second round put these to the vote. In this second round, standardized phrasing ‘it is recommended’ for at least initial 90% agreement, ‘it is suggested’ for more than 70 to less than 90% initial agreement and ‘it may be considered’ for 70% and less initial agreement was used. The grading of these consensus‐based recommendations corresponds to GRADE recommendations [9]. With the changes made in the second round, however, the % agreement indicated in the individual recommendation boxes below indicates agreement with the final phrasing. Following two successive rounds with items needing clarification, the resulting draft was then released for comments from all members of the European Society of Contact Dermatitis (ESCD; www.escd.org). Amendments were accordingly made, provided that (i) the comment was substantiated—if possible, by scientific literature—and (ii) the guideline authors found it acceptable.

Care was taken to identify scientific literature pertinent to those aspects, where it was available, by performing searches in MEDLINE (PubMed), Web of Science and other bibliographic resources. Where applicable, the search strategy and search results are described in the sections. However, for several topics, no formal studies are available; hence, the statements are based on collective expert judgement and consensus, the degree of which is indicated along each boxed recommendation.

## 2 Introduction

### 2.1 Definitions

Contact dermatitis is an inflammatory skin reaction caused by direct contact with noxious agents in the environment. The pathomechanism may involve delayed (also termed type IV) hypersensitivity (resulting in ACD) or a non‐specific response by the innate immune system resulting in irritant contact dermatitis (ICD), or may be mixed.

Contact allergy is an altered immune status of an individual induced by a particular sensitizing substance, a contact allergen. This involves a clinically unapparent sensitization phase, also called the induction phase, resulting in the expansion of a clone of allergen‐specific T cells. At this point, an individual is immunologically sensitized. In this guideline, the term contact allergy is used synonymously with contact sensitization. The substances inducing contact allergy are reactive chemicals, usually with a molecular weight of < 500 Da, but exceptionally in the range of 500–1000 Da. They are so‐called haptens that need to bind to a skin protein to become a sensitizer (allergen), except for metals that form complexes. From a chemical point of view [10], chemical sensitizers can be divided into three categories (although some chemicals might act by all pathways): haptens that can react directly (covalently) with nucleophilic side chains of amino acids, prehaptens that need a nonenzymatic (abiotic) chemical transformation such as air oxidation and prohaptens that need an enzymatic (biotic) transformation to become reactive [10]; moreover, certain chemicals are photo‐activated by UV radiation to form photo‐haptens, which may then bind to skin proteins and become photo‐allergens. In this guideline, the term allergen will be used to include haptens.

Cross‐sensitivity occurs when a person sensitized to a particular allergen also reacts to another, chemically related allergen to which he or she has not been previously exposed [10]. An individual in whom contact allergy has been induced will develop a secondary immune response following subsequent skin exposure to the same (or cross‐reacting) allergen. This process is called elicitation and will manifest as ACD. The typical morphology of acute ACD, also termed allergic contact eczema, is erythema, oedema, papules and vesicles. At a later stage, if exposure to the allergen continues, the dermatitis may become chronic and present with scaling, fissures, lichenification and hyperkeratosis.

Allergic (‘immunological’) contact urticaria and protein contact dermatitis constitute a special case, where high molecular weight allergens such as peptides or proteins induce a specific IgE response (type I hypersensitivity), which may result in both immediate urticarial (contact urticaria) and eczematous (protein contact dermatitis) lesions.

### 2.2 Indications

Patch testing is the standard procedure used to diagnose contact allergy resulting from type IV hypersensitivity. This in vivo test aims to reproduce the elicitation phase of the reaction to a contact allergen, that is, ACD. The patch test is performed by applying allergens under occlusion on the skin under standardized conditions, with readings of the test results up to 7 days later not to miss late appearing positive reactions (see Section 4). Sensitized patients must be informed about the allergens identified and the sensitization sources to avoid recurrences of ACD (see Section 11). Other types of skin tests, including those used in the investigation of cutaneous type I hypersensitivity reactions, will be briefly mentioned.

There are very rare reports that some chemical compounds such as ammonium persulfate [11] or drugs have been associated with anaphylactic reactions when patch tested in patients with strong immediate‐type hypersensitivity. These patients should undergo investigations for type I hypersensitivity. It is at the discretion of the physician to include these substances in the patch tests of these individuals after considering the risks and benefits for the patient.

Immediate testing, namely skin prick testing or prick–prick testing, can be performed in addition to patch testing for immediate contact reactions, namely for protein contact dermatitis or contact urticaria and for (hand) eczema, where such reactions can contribute to the lesions.

It is recommended that patch testing is performed in patients with:

- Suspected contact allergy, eczematous or non‐eczematous, acute or chronic, including dermatitis related to occupational exposures (100%).
- Other types of (chronic) dermatitis (eczema) not improving with treatment, for example, suspected secondary sensitization (95.2%).
- Skin and mucous membrane eruptions (including delayed‐type drug eruptions) in which delayed‐type hypersensitivity is suspected (100%).
- Clinically suspected delayed‐type sensitization to implant materials (100%).

#### 2.2.1 Postponing a Patch Test

Postponing of patch test investigations should be considered in patients with the following conditions:

- Severe or generalized active dermatitis.
- Systemic immunosuppressive treatment in high doses where withdrawal or pause is foreseen or possible.
- Patch testing during pregnancy or lactation is not known to be harmful, but most dermatologists postpone testing as a general precaution.
- Dermatitis on the upper back or other sites chosen for the application of patch tests.
- Test sites recently treated with topical corticosteroids or topical calcineurin inhibitors, because these suppress, at least to some extent, the elicitation response [12]; current practice considers 7 days sufficient for low/medium, potency [13], while for high‐potency steroids 14 days appear necessary.
- Ultraviolet (UV) radiation (sun) exposure of the test area within 2–4 weeks, and UV‐therapy within 4 weeks prior to the patch test.

These and several other factors that can affect the outcome of patch testing are discussed in Section 8.

- It is suggested not to perform patch testing during pregnancy and lactation due to limited data, although current evidence does not indicate any harmful effects (90.5%).
- It may be considered to perform a patch test in special cases, if expected benefits for the mother outweigh the theoretical risk for the child (90.5%).

#### 2.2.2 Information for Patients Prior to Patch Testing

Patients should be informed about the purpose and benefits of patch testing, how it is undertaken, and symptoms that may occur (see Section 10). It is necessary to inform on the necessity to avoid showers, wetting test sites, UV irradiation and excessive exercise or pressure on the patches that may lead to loosening of patches. Moreover, the patient should be made aware of possible symptoms such as itching and severe or late appearing reactions, and be given written information about the patch testing procedure [14].

There are various national regulations concerning patch testing. Dermatologists should be aware of the national legal frameworks within their respective countries.

## 3 Materials

Contact dermatitis textbooks and a literature search in January 2024 using combinations of the search terms ‘patch testing, contact dermatitis, contact allergy and technique’ revealed numerous publications. A large number of those dealing with technical aspects (test systems, test materials, allergens, vehicles, concentration and stability) were reviewed, and recent references were selected. The number of up‐to‐date controlled clinical experiments is limited, and many of the current standards in clinical use are based on old studies, which do not provide a high level of evidence.

### 3.1 Test Systems

Different systems are used to occlude and apply the allergens. There is no documentation proving that one test system is generally superior to the others. The choice of patch test system in a clinic is based on tradition and experience.

Usually, investigator‐loaded test systems are used. In one commonly used system, the chambers are supplied in strips of 5 or 10, and consist of small aluminium disks mounted on non‐occlusive tape that has been chosen for its adhesive properties and hypoallergenic acrylic‐based adhesive [15]. Other systems usually consist of square plastic chambers on hypoallergenic tape. The small depressions that the chambers leave on the skin when the test is removed allow for the assessment of correct application and tight fit of patches. It should be considered that test systems with aluminium disks may give rise to false‐positive reactions in patients with contact allergy to aluminium, at least to some allergens which enhance aluminium release from the discs, such as sodium tetrachloropalladate [16].

Pre‐packaged tests are also available, but only for a limited number of allergens. They currently do not fully cover the European baseline series and some allergen concentrations (effectively: doses per area) are different [17]. These pre‐packaged tests contain homogeneously dispersed allergens in standardized concentrations in a hydrophilic gel base (hydroxypropylcellulose or povidone) mounted on an acrylic‐based adhesive tape. Well‐known characteristics of these pre‐packaged tests, such as a poorer sensitivity of fragrance mix I [18, 19], formaldehyde [20] and methyldibromo glutaronitrile [21], should be considered.

Commercially available patch test allergens and systems should be of pharmaceutical quality. In some countries, only patch test allergens are commercially available that either have a market authorization or are marketable under transitional provisions or under exceptional rule by the national authorities [22].

### 3.2 Selection of Patch Test Materials

The history and examination of a patient offers clues regarding the possible sensitizers, and should guide the choice of patch test materials. Unfortunately, it is not sufficient to patch test with only those allergens that are suspected sensitizers, as unsuspected ones frequently turn out to be relevant. This is the reason why a ‘baseline series’ of test allergens should be applied in the evaluation of all patients suspected of having ACD. An allergen is suggested for inclusion in the baseline series when routine (‘consecutive’) patch testing of patients with suspected contact dermatitis results in a proportion of contact allergy to the substance exceeding 0.5%–1.0% [23, 24], when this particular allergen is common in the environment, and the results of testing are reliable and not concordant with/detected by another allergen already included in the baseline series. In particular cases, a contact allergy prevalence below 0.5%–1% can also justify routine testing, if the allergen is clinically highly relevant, or when existing knowledge of exposure is inadequate [24].

The European baseline series, as currently recommended by a taskforce of the European Society of Contact Dermatitis (ESCD), is shown in Table 1 [17]. As new allergens emerge, and some are phased out, the baseline series is dynamic and subject to continual evaluation and occasional modification, depending on improved knowledge on test preparation, population exposures, and the prevalence of contact allergy. It can be complemented to include allergens of local importance to specific dermatology departments. In most cases, application of just the baseline series is insufficient [25, 26], and additional patch test substances or series, tailored to the history and exposures of the patient, must be included.

**TABLE 1:** The current European baseline patch test series [17].

Compound (CAS number)	Concentration % (w/w)	Concentration mg/cm^2^
Potassium dichromate (7778‐50‐9)	0.5	0.2
*p*‐Phenylenediamine (106‐50‐3)	1.0	0.4
Thiuram mix	1.0	0.4
Tetramethylthiuram sulphide (TMTM) (97‐74‐5)	0.25	0.1
Tetramethylthiuram disulphide (TMTD) (137‐26‐8)	0.25	0.1
Tetraethylthiuram disulphide (TETD) (97‐77‐8)	0.25	0.1
Dipentamethylenethiuram disulfide (DPTD) (94‐37‐1)	0.25	0.1
Neomycin sulphate (1405‐10‐3)	20.0	8.0
Cobalt chloride (7646‐79‐9)	1.0	0.4
Caine mix III	10.0	4.0
Benzocaine (94‐09‐7)	5.0	2.0
Cinchocaine (Dibucaine) (85‐79‐0)	2.5	1.0
Tetracaine (94‐24‐6)	2.5	1.0
Nickel sulphate (7786‐81‐4)	5.0	2.0
2‐Hydroxyethyl methacrylate (868‐77‐9)	2.0	0.8
Colophonium (8050‐09‐7)	20.0	8.0
Parabens	16.0	6.4
Methylparaben (99‐76‐3)	4.0	1.6
Ethylparaben (120‐47‐8)	4.0	1.6
Propylparaben (94‐13‐3)	4.0	1.6
Butylparaben (94‐26‐8)	4.0	1.6
N‐Isopropyl‐N′‐phenyl‐*p*‐phenylenediamine (3085‐82‐3)	0.1	0.04
Lanolin (wool alcohols) (8027‐33‐6)	30.0	12.0
Mercapto mix	2.0	0.8
N‐cyclohexylbenzothiazyl sulfenamide (95‐33‐0)	0.5	0.2
Mercaptobenzothiazole (149‐30‐4)	0.5	0.2
Dibenzothiazyl disulphide (120‐78‐5)	0.5	0.2
Morpholinylmercaptobenzothiazole (2‐(4‐Morpholinothio)benzothiazole) (102‐77‐2)	0.5	0.2
Epoxy resin (Diglycidyl ether of bisphenol A) (1675‐54‐3)	1.0	0.4
*Myroxylon pereirae* resin (8007‐00‐9)	25.0	10.0
4‐*tert*‐Butylphenol formaldehyde resin (51732‐68‐4)	1.0	0.4
Mercaptobenzothiazole (149‐30‐4)	2.0	0.8
Formaldehyde (50‐00‐0)	2.0^a^	0.6
Fragrance mix I	8.0	3.2
Cinnamyl alcohol (104‐54‐1)	1.0	0.4
Cinnamal (104‐55‐2)	1.0	0.4
Hydroxycitronellal (107‐75‐5)	1.0	0.4
α‐Amyl cinnamal (122‐40‐7)	1.0	0.4
Geraniol (106‐24‐1)	1.0	0.4
Eugenol (97–53‐0)	1.0	0.4
Isoeugenol (97‐54‐1)	1.0	0.4
*Evernia prunastri* (oakmoss absolute) (90028‐68‐5/9000‐50‐4/68 917‐10‐2)	1.0	0.4
Sesquiterpene lactone mix	0.1	0.04
Alantolactone (546‐43‐0)	0.033	0.013
Dehydrocostus lactone (477‐43‐0) and costunolide (553–21‐9)	0.067	0.027
Sodium metabisulfite (7681‐57‐4)	1.0	0.4
Propolis	10	4.0
Methylchloroisothiazolinone (150 ppm) and methylisothiazolinone (50 ppm) (26172‐55‐4 and 2682‐20‐4)	0.02^a^	0.006
Budesonide (51333‐22‐3)	0.01	0.004
Tixocortol pivalate (55560‐96‐8)	0.1	0.04
Methyldibromo glutaronitrile (35691‐65‐7)	0.5	0.2
Fragrance mix II	14.0	5.6
Hydroxylisohexyl 3‐cyclohexene carboxaldehyde (31906‐04‐4/51 414‐25‐6)	2.5	1.0
Citral (5392‐40‐5)	1.0	0.4
Farnesol (4602‐84‐0)	2.5	1.0
Coumarin (91‐64‐5)	2.5	1.0
Citronellol (106‐22‐9/26 489–01‐0/7540‐51‐4/1117‐61‐9)	0.5	0.2
α‐hexyl cinnamal (101‐86‐0)	5.0	2.0
Hydroxyisohexyl 3‐cyclohexene carboxaldehyde (31906‐04‐4/51 414‐25‐6)	5.0	2.0
Methylisothiazolinone (2682‐20‐4)	0.20^a^	0.06
Benzisothiazolinone (2634‐33‐5)	0.1	0.04
Textile dye mix	6.6	2.64
Disperse Blue 35 (12222‐75‐2)	1	0.4
Disperse Yellow 3 (2832‐40‐8)	1	0.4
Disperse Orange 1 (2581‐69‐3)	1	0.4
Disperse Orange 3 (730‐40‐5)	1	0.4
Disperse Red 1 (2872‐52‐8)	1	0.4
Disperse Red 17 (3179‐89‐3)	1	0.4
Disperse Blue 106 (68516‐81‐4)	0.3	0.12
Disperse Blue 124 (15141‐18‐1)	0.3	0.12
Decyl glucoside (54549‐25‐6; 58 846‐77‐8; 141 464‐42‐8; 68 515‐73‐1)	5.0	2.0

*Note:* For mixes, the individual components of the mix and their concentration in the mix are given below each mix. All allergens in pet., except where indicated otherwise.

^a^ Aqua.

A number of allergens, mainly fragrances and rubber compounds, are compounded into mixes. The basic concept of using mixes of allergens instead of single allergens is to save space. However, a positive reaction to some of the mixes, such as the fragrance mixes, should normally prompt a subsequent breakdown test of its single ingredients to provide specific information to the patient. In case of a negative breakdown test a false‐positive (irritant) reaction to the mix should not automatically be concluded. In addition, when allergy is suspected, a mix should not be relied on to detect the allergy, and the individual components and additional allergens should also be tested. The mixes are frequently a compromise, in an attempt to balance sensitivity for detecting contact allergy to every single ingredient of the mix by including them in sufficient concentrations against the risk of irritation from the combination of several constituents in one test preparation. Consequently, false‐negative reactions may occur [27, 28].

### 3.3 Vehicles and Concentrations

Several hundred test allergens are available from suppliers. For the most part, these allergens are dispersed in petrolatum (white soft paraffin), and are supplied in labelled syringes with the name and concentration of the substance on the label, together with an expiry date. Petrolatum (pet.) is inexpensive, practical, gives good occlusion and can be mixed thoroughly with most substances. However, the choice of vehicle is important, and some substances are, due to physico‐chemical properties, better tested in solution, for example, in water or ethanol.

Test concentrations have been mainly selected on the basis of experience to elicit an allergic response in those previously sensitized and to cause no positive reaction in those who are not allergic. For these allergens, patch test sensitization (‘active sensitization’) is considered to be extremely rare, particularly when recommended test doses are followed [29]. For convenience in clinical practice and standardization, groups of test allergens are arranged into test series.

Not all allergens are commercially available as test preparations and are therefore not covered in the baseline series or in special test series. To fill these diagnostic gaps and to allow for detection of novel causative allergens, it may be necessary to patch test patient's own materials [30, 31] (see Section 6) or to make up constituent ingredients of incriminated products for patch testing. For this purpose, it is sometimes necessary to obtain these ingredients directly from the manufacturer. Care should be taken to use the appropriate concentration, that is, dose in mg or μL/cm^2^, and vehicle [32]. Moreover, potential issues regarding purity, original concentration and reliability of materials and information obtained from the manufacturer should prompt extra caution.

### 3.4 Storage and Stability

Patch test materials should be stored at 4°C (or colder, as required according to specific instruction of the manufacturer) and be protected from light. To preserve the homogeneous distribution of allergens within the vehicle, horizontal storage is required. Some contact allergens with high vapour pressure, such as some fragrance chemicals, acrylates and isocyanates, are unstable and require more frequent renewal, strict storage conditions and should be loaded in test chambers just prior to application [32, 33, 34, 35, 36]. Glutaraldehyde in pet. and formaldehyde in aqueous solution are also subject to instability and deterioration. It is important to respect expiry dates [37, 38].

**Conclusion**: Patch test materials should be stored horizontally at 4°C, protected from light. Special characteristics of the patch test allergens (volatility and stability) must be considered.

## 4 Technique

### 4.1 Dosing of Chambers

The critical factor for sensitization and elicitation of contact allergy is the ‘dose per unit area’ [39]. Therefore, it is important for the dose of allergen to be standardized for each type of test chamber (Table 2) [40, 41, 42]. For example, for 8 mm Finn Chambers, 20 mg of each allergen in pet. (∼40 mg/cm^2^) is squeezed from the syringe into the chamber such that it fills the well of the disk but does not extrude when the patch is applied to the back [43, 44]. For water‐based allergens, small filter papers are placed in the well, unless the chamber is already equipped with filter paper, and these will hold ∼15 μL of liquid. The dosing of liquids by use of a micropipette is strongly recommended [45]. Besides the micropipette technique, there are two other major ways to apply a test solution onto a chamber. In the drop technique, a drop of solution is placed on the chamber by squeezing the plastic bottle containing the test solution. In the drop and wipe technique, a drop of test solution is placed on the filter paper of a test chamber by squeezing the container. Before testing, the excess solution is wiped off with a soft tissue. A study comparing the three techniques showed that the micropipette technique had the best accuracy and precision [45]. If the same amount/volume of a test preparation is applied all the time with the same test technique (same area of skin) and occlusion time, it is appropriate to use concentration as a dose parameter. For most allergens, pet. is an appropriate vehicle, as it is stable and seems to prevent/diminish degradation, oxidization and polymerization, but not evaporation, of the incorporated allergen [35, 46, 47]. Dosing of pet.‐based allergens needs training and experience to keep the variation within a limited range [48, 49]. When other test chambers are used, the same dose/unit area skin can be used.

**TABLE 2:** The optimal doses of pet. and liquid preparations, respectively, in different, commonly used chambers are as follows [4].

	Liquid		Preparation in pet.	
	μL	μL/cm^2^	mg	mg/cm^2^
Finn Chamber (diameter 8 mm; area 0.5 cm^2^)	15	30	20	40
Van der Bend (area 0.64 cm^2^)	20	30	25	40
IQ Ultra (area 0.68 cm^2^)	20	29	27	36

*Note:* Doses for all other chamber types can be calculated.

Generally, pet.‐based patch test substances should be loaded into the chambers shortly before application of the patches (no longer than a few hours), liquids and some volatile pet.‐based substances (e.g., fragrance materials and acrylates) at the time of application.

It is recommended to dose liquids with a micropipette (90.5%).

### 4.2 Anatomical Site of Patch Test Application

For practical reasons, the convex surface of the upper back is chosen. The back offers a relatively flat surface for good occlusion, and is usually large enough for application of the necessary number of patch test substances. It is less often affected by skin diseases, is not regularly exposed to sun, and is less prone to scratching. Sometimes, the outer surface of the upper arms or thighs can also be used if the surface on the back of the patients is insufficient or cannot be used for other reasons, for example scars, acne or large tattoos.

There are known variations in reactivity of the skin between different anatomical regions. For example, the forearm is less sensitive than the back to elicitation of contact allergy to nickel [50]. When comparing the sensitivities of various skin sites in the repeated open application test (ROAT), Hannuksela [51] found that the lower arm was less sensitive than the upper arm, and that the skin of the back was most reactive (see Section 5). Some studies showed higher reactivity of the upper back, especially when laser Doppler flowmetry was used for evaluation [52], than of the lower back, but later studies have not confirmed such a difference [50, 53]. In a recent study, there were no differences in patch test reactions and sites of application on the upper back when using a high level of standardization of the patch test technique [54]. For comparability and standardization, it is important to always use (if possible) the same anatomical site.

It is recommended to use the upper back for patch testing. The outer surface of the upper arms or thighs can be used if the back is not suitable for patch testing, or is fully used already (100%).

### 4.3 Occlusion Time

Occlusion time is the duration of application of the patch test allergen to the skin. Exposure of the outer surface of the horny layer to the haptens is obtained with an occlusive patch test chamber system. Penetration is enhanced by occlusion; the quantitative aspects of this process are not fully known. However, the penetration of substances and the process of enhanced penetration with the help of occlusion (which, among other factors, increases the hydration of the skin and most likely facilitates the penetration of less lipophilic or mainly hydrophilic substances) vary considerably between different chemical substances. The currently used occlusion time established for patch testing is a practical compromise, which makes it possible to patch test with many different substances at the same time.

It has been shown for nickel that 2 days of occlusion gives a higher frequency of positive reactions than 1 day of occlusion [55]. However, it has also been shown for nickel that shortening the occlusion time can be compensated for by a higher test dose [56]. Interestingly, a recent study with oxidized terpenes in more than 5000 patients at different German, Swiss and Austrian clinics demonstrated a significantly higher frequency of reactions using occlusion for 2 days as compared to 1 day [57]; however, the observed reaction pattern in the study suggests that both test preparations used have irritant potential with an increased risk of false‐positive reactions. Isaksson et al. compared 5, 24 and 48 h of occlusion for several dilutions of budesonide in allergic subjects, and found that 48 h of occlusion gave the highest number of positive responses [58].

Neither the literature study of Manuskiatti and Maibach [59], nor the data of Brasch et al. [60] showed evidence for a general superiority of 1 day versus 2 days of occlusion. Explanations might include that the testing with two occlusion times has not been in the same patients [60], no defined doses in mg/cm^2^ or μL/cm^2^ have been used, or a too low power [59]. Obviously, to draw robust conclusions on which occlusion time to be used, the testing with the two occlusion times should be performed simultaneously in the same patient. Such a study was recently reported demonstrating the superiority of 2 days versus 1 day of occlusion [61]. Longer application periods are not recommended.

In studies on PPD‐allergic subjects, it was shown that, with longer occlusion time, lower concentrations of PPD were sufficient to elicit a positive response [62]. In cases of strong contact allergy to PPD, 30 min of application of PPD 1% pet. was sufficient to elicit positive responses. This was not the case for those patients who showed lower reactivity. For some contact allergens (in the particular case, a photocontact allergen), for example ketoprofen [63], a much shorter occlusion time (1 h) than 48 h seems to be as effective as the traditional occlusion period. If an extremely strong patch test reaction is expected, a lower test concentration or shorter occlusion time can be used.

It is recommended to employ an occlusion time of 2 days (100%).

### 4.4 Reading Times

**FIGURE 1:**
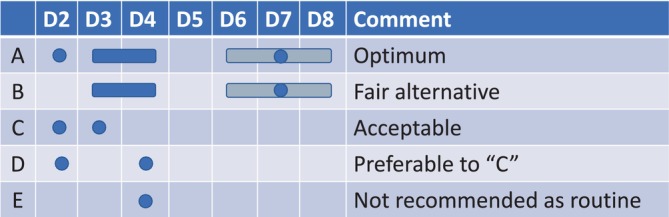
Common reading schemes. D, days after start of patch test application.

After test application at Day 0 (D0) and allergen exposure for 2 days, the patch test chambers are removed. The following reading times are often used in practice (Figure 1):

  A D2 and D3 or D4 and around D7 (optimum): usually at D2, after 15–60 min, allowing for the resolution of pressure effects, the test reaction is read for the first time [2, 64, 65, 66, 67, 68, 69, 70]. A reading at D3 or D4 is obligatory [65, 68]. A reading between D5 and D10 is necessary for at least some allergens, for example corticosteroids, gold, acrylates and aminoglycoside antibiotics, for which 7%–30% of contact sensitizations will be missed if a reading around D7 is not performed in addition to the reading on D3 or D4 [71, 72, 73, 74, 75]. Also in psoriasis patients allergic patch test reactions (to nickel) were found delayed as compared to non‐psoriatic patients, with a maximum intensity after 7 days [76].
  B D3 or D4 and around D7 (fair alternative): in some countries, the first reading is on D3 or D4.
C,D D2 and D3 or, preferably, D2 and D4 (acceptable): if organizational circumstances dictate, two readings as above will allow for the diagnosis of the vast majority of contact allergies to most allergens, with, however, a risk of false‐negative results, particularly for some allergens (see above).
  E D4 only (not recommended as routine): in a study in which patch tested individuals were read several times in the range D2–D9, the single day that traced most contact allergy was D4, but to trace all cases of contact allergy two readings on D4 and D7 were required [71]. A D2 reading as the only reading is not appropriate [77].

Owing to geographical or organizational circumstances, the reading times may vary. In exceptional cases, if a physical reading on D7 is not possible, photos may be used as a substitute, after careful preparation and instructions, and conscious of some associated error [78].

It is recommended to perform at least two readings of the patch test reactions. Ideally, readings are performed at D2, D3 or D4 and D7 (95.2%).

### 4.5 Morphology

The reading of patch test reactions is based on inspection and palpation of the morphology (erythema, infiltrate, papules and vesicles) as shown in Table 3.

**TABLE 3:** Reading criteria.

Symbol	Morphology	Interpretation
−	No reaction	Negative reaction
?+	Faint erythema only	Doubtful reaction^a^
+	Erythema, infiltration, possibly papules	Weak positive reaction
++	Erythema, infiltration, papules, vesicles	Strong positive reaction
+++	Intense erythema, infiltration, coalescing vesicles	Extreme positive reaction
IR	Various morphologies, for example, soap effect, bulla, necrosis	Irritant reaction

^a^ Irritant or allergic.

Morphologically positive patch test reactions are those that show +, ++ or +++ morphology on at least one reading. Doubtful reactions (?+) can sometimes be clinically relevant and important for the individual patient [79, 80], for example, to neomycin sulphate. They may need further work‐up (e.g., repetition of the patch test with several concentrations/serial dilutions, or use test) [81]. It is important to pay attention to the fact that a positive use test demonstrates that the doubtful reaction (?+) is clinically relevant as it has resulted in a contact dermatitis but it does not tell whether the doubtful reaction represents an irritant or weak allergic reaction [81, 82]. Well‐demarcated erythematous reactions are often seen with fragrance mix, *Myroxylon pereirae* resin, caine mix III and thiuram mix. Skin of colour will show erythema as darker‐appearing than on white skin, which needs to be considered.

Recently, inter‐observer variability has been identified in the discrimination between doubtful and irritant reactions [42, 80, 83, 84]. Continuous standardization and reading training is advisable [42, 83, 84]. Although recent Deep Learning approaches towards automated patch test reading from images have shown promising results [85, 86, 87], machine learning tools have not yet become standard of care in the reading of patch tests.

Different types of irritant reaction have been described (Table 4) [2]. Sharp‐edged margins and fine wrinkling of the surface of the test area point towards irritant reactions. Reactions appearing purpuric are commonly caused by metal salts, for example cobalt chloride. Pustular reactions are seen mainly with non‐noble metals such as chromium, cobalt and nickel. In special cases, pustular reactions may reflect contact sensitization [90], particularly when they are non‐follicular and evolve from vesicles. For the interpretation, it is necessary to keep in mind that, besides their properties as patch test allergens, many patch test chemicals also have some irritant potential [91], which is more predominant for some allergens (e.g., benzoyl peroxide, phenyl mercuric acetate, propylene glycol, benzalkonium chloride, octyl gallate, cocamidopropyl betaine and 1,3‐diphenyl guanidine), frequently resulting in weak erythematous (doubtful) test reactions [92, 93, 94]. A relevant factor for the assessment of patch test results is the particular skin sensitivity and irritability of the individual tested at the time of patch testing. At times of individually increased skin irritability, more non‐specific doubtful test reactions may occur. An irritant control patch test is performed in some centres (e.g., sodium lauryl sulphate 0.25% aq.) [95] where it is considered to assist in the interpretation of weak reactions to allergens. The value of such a ‘control’ has not been unequivocally proven. Table 4 includes a differential diagnostic approach to irritant and positive patch test reactions [2, 6, 58, 88, 89].

**TABLE 4:** Differential diagnosis between irritant and allergic patch test reactions.

Clinical feature	Allergic patch test reaction	Irritant patch test reaction
Erythema	With infiltration	Without infiltration
Reaction in the test area	Reaction extending beyond the test area, with no sharp margins (rounding at the corners when testing with square‐shaped chambers)	Reaction confined to the test area, with sharp margins^a^
Bullous/erosive reaction	Bullae evolving from vesicles	Direct formation of bullae/erosion, without evolving from vesicles
Pustules	Non‐follicular pustules evolving from vesicles on a background of erythema and infiltration	Follicular pustules, without evolving from vesicles. No erythema or infiltration
Papules	With erythema and infiltration in the test area, with/without vesicles	Isolated follicular papules
Edge effect (Reaction confined to the periphery of the patch test area.)	Primarily with liquid allergens and corticosteroids [58], evolves into a positive reaction during subsequent readings. Special patterns: Passe‐partout effect [88], extra‐edge effect [89]	Physically, as pressure effect from the rims of the test chambers, or chemically, from the accumulation of irritant chemicals at the margins of the chamber
Purpura	Not applicable	Petechial/purpuric macules, best known for cobalt chloride
Soap effect	Not applicable	Shiny and wrinkled skin surface
Temporal pattern	Plateau or crescendo^b^	Decrescendo^b^
Delayed/late onset	Occasionally	No
Symptom at the test site	Pruritus/itching	Burning/stinging

^a^ This morphology is not necessarily indicative of an irritant reaction. Certain contact allergens (e.g., methylchloroisothiazolinone/methylisothiazolinone) or dilutions of many sensitizers may also display reactions of this morphology.

^b^ This concerns readings on D2 and D3/D4 and D7, respectively.

After the final reading of patch test reactions, a conclusive interpretation is mandatory concerning the relevance of the test reactions in the respective case with regard to the patient's history, exposure and clinical course (see Section 7). When there is a positive reaction to patient‐supplied chemicals/products and non‐established contact sensitizer patch test preparations, the possibility of false‐positive reactions should be considered. In these cases, patch testing in controls might be needed to confirm/rule out allergenicity as the cause of the positive reactions, if possible.

**Conclusion**: The patch test is scored according to morphology. An allergic patch test reaction to a well‐established sensitizer tested according to guidelines is generally defined as a reaction that fulfils the criteria of at least a + reaction.

## 5 Other Techniques

### 5.1 Semi‐Open Test

Goossens suggested the use of the semi‐open test mainly for testing patient‐supplied products with suspected irritant properties, for example shampoos, detergents, paints, varnishes, cooling fluids, pharmaceuticals and some cosmetics [96]. Before performing this test it is mandatory to confirm the patient usually tolerates direct contact with the material with no burning/harm, or to evaluate at least the pH of the product to avoid chemical burns from acids or alkali (see also Section 6).

For the semi‐open test, a small amount (∼15 μL) of the product is applied with a cotton swab on an area (1 cm^2^) of the skin, allowed to dry completely, checked for signs of contact urticaria and then covered with permeable tape.

Readings are performed in the same way as for patch testing. Immediate reactions may appear after 20–30 min as a sign of contact urticaria, and dermatitis reactions may develop at D2–D4. This is a diagnostic tool that requires some experience for interpretation.

### 5.2 Open Test

The same precautions as with the semi‐open test (previous acute contact tolerated, pH checked) shall be taken. In the open test, a product, ‘as is’ or dissolved in water or some organic solvent (e.g., ethanol or acetone), is dripped onto the skin and allowed to dry. No occlusion is used. The usual test site is the volar forearm, but it is less reactive than the upper back or the upper arm. An open test can be useful as the first step for testing substances or products such as those brought by the patient (see Section 6). Readings are made at regular intervals during the first 30–60 min after application, in order to detect immediate reactions, including urticaria. A second reading should be performed at D3–D4. A negative open test result can be explained by insufficient penetration but indicates that one may proceed with an occlusive patch test.

### 5.3 Strip and Scratch Patch Tests

Removal of a substantial part of the horny layer prior to application of patch tests will reduce the epidermal barrier and thus enhance sensitivity. To this end, a standardized methodology has been developed [97], which has been found to not only decrease the epidermal barrier, but also to create a pro‐inflammatory milieu which probably contributes to the increased sensitivity of this test [98]. The authors concluded, based on acceptable reliability comparable with conventional patch tests, that application in routine clinical practice can be recommended especially if the conventional patch test result is presumably false‐negative [99]. The reaction profiles (‘reaction index’ and ‘positivity ratio’) of baseline series allergens have been found largely unaffected by prior tape stripping, pointing to safe applicability across the baseline series allergens [100].

A more drastic reduction of epidermal barrier integrity is the aim of the scratch patch test. This has been found useful mainly in cases with cutaneous adverse drug reactions (CADR), for example, to imatinib [101], brimonidine tartrate (where scratch patch testing demonstrated markedly superior sensitivity compared to a simplified strip patch test employing only 10 repeated tape applications rather than standardized methodology [97]) [102], and other drugs. Moreover, a diagnosis of contact allergy to 2‐octyl cyanoacrylate (Dermabond) could be established only by using this method, closely mimicking clinical exposure in the case of this surgical glue [103].

### 5.4 Repeated Open Application Test

The ROAT was developed by Hannuksela and Salo [104]. It is a standardized exposure test mimicking a use situation that aims at eliciting ACD in the test area. With this method, it is possible to clarify the clinical importance of selected patch test reactions. In some cases, contact allergy to a product can only be proven with this technique, namely for chemicals we are exposed to in low concentrations and that need to accumulate in the skin upon repeated exposures to reach the threshold for eliciting ACD. The ROAT may be useful both in experimental research studies [105] and in clinical routine.

#### 5.4.1 Methodology

Test solutions, either commercial products or special test substances, are applied twice daily for up to 10 days [106] to 2 weeks (but sometimes for up to 4 weeks) on the flexural (volar) aspect of the forearm near the antecubital fossa. The size of the test area is usually 3 × 3 to 5 × 5 cm, and the amount of test substance should be sufficient to cover the test area. The applications continue until a reaction develops or until the end of the selected exposure period. It may be advisable in selected cases to include a control substance on the contralateral arm, and the ROAT may also be performed in a blinded fashion.

#### 5.4.2 Reading and Evaluation

A positive response in the form of ‘eczematous’ dermatitis, often starting with follicular papules, may appear after a few days or later, depending on dose/area, matrix effects and individual elicitation thresholds. However, a negative ROAT result after 1–2 weeks does not exclude a relevant contact allergy. Therefore, in cases with high suspicion, extended application periods of 3–4 weeks may be important, in order not to miss late‐appearing reactions. Johansen et al. [107, 108] developed a scale for the evaluation of ROAT responses (Table 5).

**TABLE 5:** Modified scale for reading repeated open application test results.

Score points per criterion	0	1	2	3	4
1. Involved area of application	0	1%–24%	**25%–49%**	50%–89%	90%–100%
2a. Erythema (Involvement)	None	**Spotty**	Homogeneous		
2b. Erythema (Strength)		**Weak**	Medium	Strong	
3. Papules	None	**1–5**	5–10	> 10	Homogeneous infiltration
4. Vesicles	None	1–5	5–10	> 10	Confluent

*Note:* The scale applies to a 3 × 3‐cm application area; for larger areas, the numbers of papules and vesicles need to be proportionally scaled up. The minimum requirement for a positive test is marked in bold, equivalent to that of 5 points as used in research studies [107, 109].

Each variable (1–4) must be given a score from 0 to 2 or to 3 or 4 (see Table 5). A positive reaction is characterized by erythema and infiltration as represented by papules, as a minimum, usually accompanied by pruritus or itching at the ROAT site, and the reaction should cover altogether at least 25% of the test area. The single scores are added, and a positive reaction will range from 5 points to a maximum of 17 points.

**Conclusion**: The ROAT is used to clarify the relevance of selected positive and doubtful patch test reactions by testing (leave‐on) cosmetics, topical drugs and other suitable formulations.

As a variant of a ROAT, repeated application of pieces of glove(s) suspected to have caused ACD has been suggested and termed glove repeated application test (GRAT) [108]. A piece of the glove is held by an adhesive strip during 10 consecutive nights on the same area of the volar forearm. For disposable gloves, a new piece was used each night, otherwise, the same piece of glove was reused. Positive reactivity was defined according to above‐mentioned criteria.

Yet another type of ROAT is a ‘(provocative) use test’. This involves application of a commercial product, often a cosmetic product, containing a diagnosed contact allergen. The use test, with application according to normal habits of the patient, has occasionally been used in clinical research studies [110, 111], and not infrequently in case studies, and may be useful to sensitively elucidate clinical relevance, especially if used on formerly affected, but healed, skin sites.

- It is recommended that the less standardized tests (semi‐open, open, strip and scratch patch test) are undertaken only by clinicians experienced in patch testing who fully understand the potential hazards of the applied substances/products (95.2%).
- It may be considered to perform the ROAT to confirm relevance of a contact allergen with products or dilutions intended for repeated skin exposure (100%).

### 5.5 Photopatch Testing

Photopatch testing is mainly indicated in the study of photoallergic contact dermatitis, where UV exposure is necessary to induce the hypersensitivity reaction. It can also be helpful in the study of any dermatitis on photo‐exposed areas or photosensitivity resulting from the use of systemic drugs [112]. For photopatch testing, a duplicate set of allergens is prepared and applied on two corresponding areas of the back. After 1 or 2 days of occlusion, one set of tests is irradiated with 5 J/cm^2^ of UVA while the other is completely shielded from light and kept protected until further reading. A retrospective study comparing 1–2 days of occlusion before irradiation showed the longer exposure time to be more sensitive; however, it was concluded that systematic studies were needed for definite conclusions to be drawn [113]. The 1‐day occlusion/reading is usually used in photobiology clinics for combination with the readings of phototests to determine minimal erythema dose (MED), whereas, traditionally, contact dermatitis clinics use 2 days of occlusion before irradiation. Readings should be performed before and immediately after irradiation and at least 2 days thereafter, and if possible, also later. Grading of the reactions should follow the general rules of patch test readings but, for result interpretation, it is necessary to compare reactions between the irradiated and the non‐irradiated sites. A positive photopatch test result occurs when there is no reaction at the non‐irradiated site and a positive (+ to +++) reaction at the irradiated site. Positive reactions in both sets of tests represent contact allergy (Table 6).

**TABLE 6:** Diagnosis of contact allergy versus photo contact allergy based on the photopatch test.

Non‐irradiated	Irradiated	Final diagnosis
Negative	Positive	Photo contact allergy
Positive	Positive	Contact allergy
+	++	Photo aggravation
++	+++	

At present, the recommended European photopatch baseline series includes mostly UV filters of different chemical families, non‐steroidal anti‐inflammatory drugs (NSAIDs) and a few photosensitizers known for a long time [114]. A more extended photopatch test series may be used, and any product suspected of being implicated in the reaction, particularly the patient's sunscreen(s), should also be photopatch tested. In a photosensitive patient, it is recommended to first determine reactivity to UV light (phototests performed on the day of application of the patches), and the UV dose for irradiation of the test site should be only 75% of the patient's minimal erythema dose [115]. In exceptional cases with high suspicion, UVB can additionally be used to irradiate one set of allergens following evaluation of the individual UVB MED. In this case one set of allergens is irradiated with 50%–75% of the calculated individual UVB MED.

## 6 Patch Testing of Patients' Own Materials

The textbook chapters in the references provide more detailed information on this subject [30, 31, 32]. The information in this section is based on practical observations and empirical evidence, as no experimental data exist in this area.

Patch testing with patients' own products is especially important in occupational dermatology, because standardized commercial patch test substances of many occupationally used chemical compounds are lacking. At least 5000 contact allergens are known, but only several hundred commercially available allergen preparations exist and not all of them are in fact readily available in all places [22]. The number of allergens in an ordinary test laboratory is usually much lower. Thus, not all possible contact allergies can be identified with commercial allergens, and testing with patients' own products is necessary. Moreover, our environment is constantly changing, and workers and consumers are exposed to new chemicals, some of which are allergens. Routine test substances will not identify new allergens. Testing with patients' own products is the only way of finding new allergens in the clinic. Efficient testing of patients' own substances requires experience and well‐trained staff. In patients with suspected occupational ACD, patch testing of workplace materials is particularly important [32, 116, 117]. Previously known allergens can be found in new types of products; that is, testing with patients' own materials may reveal previously unknown sources of sensitization or elicitation of ACD. In addition, patch testing with patients' own materials often helps in the assessment of the clinical relevance of an allergic reaction to tested commercial allergen preparations: for example, when a cosmetic product induces an allergic reaction and the patient also reacts to some of the ingredients labelled on the product, the allergen is probably the cause of the patient's problems. A negative result with a patient's own product does not exclude contact allergy to some of its components.

When testing patients' own materials, patients should bring samples and information on the suspected products' ingredients and hazard properties such as safety data sheets (SDSs), lists of ingredients on the packages (e.g., International Nomenclature of Cosmetic Ingredients [INCI] lists), or other types of information leaflets. Similar information may be found on the internet (the validity to be checked) and can be requested from manufacturers. Regulation (EC) No 648/2004 of the European Parliament and of the Council on detergents states that manufacturers must provide the ingredient list of detergents to medical personnel upon request [118]. SDSs provide only limited information, and not all sensitizing components may be listed. Totally unknown substances should never be tested due to safety reasons: necrosis, scarring, pigmentation/depigmentation and systemic effects caused by percutaneous absorption may appear. Extremely hazardous chemicals (e.g., corrosive substances such as strong acids and alkalis, and chemicals highly toxic via skin) or products without sufficient information should not be tested.

### 6.1 Patch Test Concentrations

The choice of test concentration is based on the characteristics of the product (skin irritant components, sensitizing components, pH, etc.). Those ingredients of the product that are available as commercial test substances should also be tested. As far as the concentrations of ingredients in the product are known, the dilution of the product should be such that none of the ingredients is above the recommended test concentration for this substance [32]. As a drawback, this may lead to insufficient concentrations of other ingredients in the test preparation (false‐negative results). Contact dermatitis or occupational dermatology textbooks contain recommendations on test concentrations [30, 31, 32].

If the number of suspected materials is small, using a dilution series of the suspected material is recommended. When putative new allergens are investigated, retesting with a dilution series down to negative concentrations is of utmost importance. Allergic‐appearing reactions that extend to very low concentrations strongly support the allergic nature of the reaction. The strength of allergic reactions gradually diminishes with decreasing concentration, whereas a false‐positive, that is, irritant reaction may vanish abruptly when the concentration is lowered. From a scientific point of view, to rule out an irritant reaction, the suspected allergen should also be tested at least on five, preferably on 20 unexposed control patients (e.g., in clinical trials; under routine test conditions outside clinical trials, ethical considerations and legal framework of the respective country have to be complied with). Test reactions on unexposed patients indicate an irritant rather than allergic reaction.

Identification of a new allergen often requires several test sessions. The components of the product are tested in the second phase, preferably with a dilution series down to negative concentrations (often ppm level). The concentration should not exceed the recommended test concentration for the type of product or chemical group (e.g., acrylates, 0.1%; methacrylates, 1%–2%). A low threshold concentration itself supports the allergic nature of the reactions, as irritant reactions to very low concentrations are rare. Guidance on dilutions and vehicles for various products is available in the following section and in the literature [30, 31].

Recommended test concentrations for different product categories:

- Leave‐on cosmetics, protective creams and topical medicaments can usually be tested ‘as is’, because they are intended to be applied to the skin as such. A negative test result does not exclude contact allergy to the product, for example, the concentration of an ingredient in the products may be too low, corticosteroids may have an anti‐inflammatory effect, etc.
- Rinse‐off cosmetic products such as liquid soap, shampoos and shower gels can be tested at concentrations of 1%–10% in pet. or aq., depending on the formulation.
- If necessary, household cleansers and detergents can usually be tested at 1%–10% in pet. or aq. **Caution:** pH needs always to be checked before dilution and if needed, adjusted to 4–9. Of note, many household cleaners are strongly alkaline, and many bathroom cleaners acidic.
- Metalworking fluid (MWF) should be patch tested with an unused concentrate diluted at the patch testing clinic. Water‐based fresh metal‐working fluids can usually be tested 5% aq. or in a dilution series of 10%–3.2%–1% pet.
- Addition of biocides during the use cycle of the MFW is common practice [119]. Parallel testing of the unused and used MWF from the machine has been recommended [119]. However, used MWFs represent mixtures of unknown composition. They may contain dissolved metals, small metal particles, lubricants and added biocides. **Caution**: They should not be routinely tested unless suspected to contain dissolved metals or added biocides that cannot be obtained separately. Preferably, one should obtain MWF additives from the workplace and test them separately. Used water‐based MWFs of use concentration 4%–8% in the machine can be tested as they are, but pH adjustment may be needed. If the use concentration is ≥ 8%, dilution down to 5% is necessary [119].
- Non‐emulsifiable MWF (neat oils or straight oils) are tested at a concentration of 50% in olive oil [119], or 50%–20% pet.
- 2‐component epoxy products: the epoxy resin part may usually be tested 1%–2% pet. and the hardener part (polyamine hardeners) 0.1%–1% pet [120, 121, 122]. **Caution**: Epoxy hardeners are skin irritants and some are corrosive. The safety of testing must be separately evaluated for each product.
- 2‐component polyurethane products: The part that contains isocyanates (normally the hardener part) may usually be tested as 2%–5% pet [123]. **Caution**: The safety of testing must be separately evaluated for each product.
- Acrylate products: [124] Methacrylates (e.g., in glues, dental resins, gel nail products) may usually be tested (if this is deemed necessary in view of non‐explanatory test results with commercially available (meth‐)acrylates) 1%–2% pet., acrylates 0.1% pet. and cyanoacrylate glues ‘as is’, dried in the test chamber, or 10% pet. [124]. A scratch patch test may be necessary to diagnose cyanoacrylate contact allergy [103]. **Caution**: The test concentration must be separately evaluated for each product, bearing in mind that products may often contain both methacrylates and acrylates.
- Solid materials (paper, textile, plastic, rubber, metal specimens of suitable shape, wood dust, etc.) can usually be tested as they are, moistened with water or organic solvents.
- Powdery materials, ground dust, scrapings or small cut pieces (if not sharp) can be tested mixed in pet. or moistened with water.
- Larger pieces (glove material, textiles, etc.) that are not too thick can be cut into a test chamber or tested semi‐open, covered with surgical test tape without a chamber. Tests can be false‐negative if insufficient amounts of the allergen are released onto the skin. Thus, pieces of, for example, rubber or plastic gloves may be tested in parallel moistened with ethanol or acetone to enhance test sensitivity [31, 125]. Pressure effects and mechanical trauma caused by sharp particles must be distinguished from allergic reactions.
- Plants: Certain plant allergens are commercially available (e.g., sesquiterpene lactones, primin, tulipaline A and diallyl disulfide). **Caution**: Some plants are irritants, and some may actively sensitize (i.e., cause sensitization by the patch test, see Section 10). Fresh or dried plant material may be tested ‘as is’, provided that the botanical identity is known and the risk of strong irritancy or test sensitization is considered low. It is advisable to test the plant parts (flower, leaf or stem) separately, moistened with water and with ethanol (two patch tests for each), because some allergenic components may be water‐soluble and others ethanol‐soluble. **Caution**: Tropical woods can also be strongly irritating and actively sensitize. Anecdotal evidence indicates infections may develop at the patch test site when testing with fresh plants [3, 126]. Cooperation with a specialized botanist and consultation of available references [127, 128] is recommended.
- Test concentrations of medicinal products for systemic use follow their own rules and consultation of available references is recommended [129, 130].

### 6.2 Vehicle

The choice of vehicle depends on the characteristics of the product, solubility and pH. Petrolatum is non‐irritant, widely used (also in commercial preparations) and suitable for most substances or mixtures; for example, various oils mix very well with pet. Most chemical products for occupational or consumer use, including detergents and water‐insoluble chemicals, are best diluted in pet., but water, acetone, ethanol, olive oil and methyl ethyl ketone are other alternatives [32]. When using water, it is important to check the pH before testing and adjust it to 4–9 if needed. For testing more alkaline or acidic substances, the use of buffer solutions is recommended. Acid buffer is used for alkaline products (pH > 9) and alkaline buffer for acid products (pH < 4), with monitoring of the pH [131].

### 6.3 Extracts and Chromatograms

The use of ultrasonic bath extracts is an alternative for testing solid materials such as gloves and plants. Small pieces of the material are cut and placed in water or organic solvent (usually acetone or ethanol), extracted in an ultrasonic device and filtered [132]. Patch testing with thin‐layer chromatograms (TLC) can be valuable if ingredients are not known or cannot be separated from the material. However, preparing TLCs for skin testing requires expertise [133].

### 6.4 Preparation of the Test Material

It is advisable to use disposable containers, syringes, stirrers and spatulas as well as appropriate protective gloves and goggles when preparing test substances. Solid materials in crystal or powder form should be ground in a mortar. Solids and viscous materials are measured by weighing and liquids by weighing or by using pipettes and syringes. The allergen is weighed or pipetted to a mixing cup, and vehicle is added to gain the desired concentration. For example, 0.1 g of material is weighed and filled up to 10 g with pet. to acquire 1% (w/w) dilution in pet. Thorough mixing is important for a homogeneous distribution of the allergen in the vehicle. Downward dilutions can be prepared from these preparations. The test preparations should be stored in a refrigerator in tightly closed containers or syringes.

- It is recommended that the products' composition is assessed by reading safety data sheets, ingredient lists, information from the manufacturer and other data. (100%).
- It is recommended to test individual components of the patients' own products separately if possible (95.2%).
- It is recommended to check the pH, if necessary, and adjust to 4–9 before testing (95.2%).
- It is recommended that the test concentration of any individual ingredient in a product to be tested does not exceed the recommended test concentration for this substance (100%).
- It is recommended to usually test leave‐on cosmetic preparations, protective creams and topical medicaments ‘as is’, unless the composition indicates otherwise (95.2%).
- It is suggested to usually test rinse‐off cosmetic products such as liquid soap, shampoos and shower gels at concentrations of 1%–10% in aq./pet. unless the composition indicates otherwise (90.5%).
- It is recommended to test suitable solid materials which had come into contact with the patient's skin ‘as is’ (91.7%).
- It is not recommended to test unknown materials and products without sufficient information regarding their composition and safety (95.2%).

It is important to remember that a negative result with the product does not exclude contact allergy to some of the product's components.

## 7 Final Evaluation: Clinical Relevance and Diagnosis

The literature was searched by the use of PubMed with the following search terms: ‘(guideline OR recommendation) AND clinical relevance AND (patch test OR patch testing OR contact dermatitis)’; ‘(clinical relevance OR relevance assessment) AND (patch test criteria OR allergy criteria)’; ‘diagnosis AND clinical relevance AND patch test’. Relevant publications were retrieved in March 2025. Textbooks were checked manually. Additionally, relevant references from the found articles were checked, and if deemed appropriate, included for review.

### 7.1 The Concept of Clinical Relevance

A morphologically positive patch test reaction to a substance at a non‐irritant patch test concentration is a sign of contact allergy, that is, sensitization to the substance in question has occurred. The next step is to determine how the patient may have been exposed to the allergen and, subsequently, evaluate whether the patient currently has, or in the past has had, any pertinent clinical symptoms (i.e., ACD) caused by sufficient exposure to the substance [134]. Thus, diagnosing ACD involves a process with two major steps: (i) demonstration of contact allergy and, following exposure assessment, (ii) the establishment of clinical relevance. Relevance for positive patch tests is determined at the final reading. A positive patch test to an allergen is of clinical relevance if [134]:

1. Sufficient exposure to that sensitizer occurs (or has occurred), taking into account the route, dose, duration and frequency of exposure and individual factors AND.
2. If a dermatitis exists (or existed), which, by considering its type (clinical features), anatomical site(s) and time course, is partially or completely understandable and explainable with regard to that exposure.

‘Sufficient’ exposure to allergen is an important nuance because many exposures in our environment are by themselves not always sufficient to elicit dermatitis, even in sensitized individuals. However, high dose exposure, or repeated, cumulative or combined low exposure (e.g., with other allergens or irritants), or when exposure occurs on compromised (damaged) skin, or with co‐factors such as occlusion, friction and sweating, may result in dermatitis [64].

**FIGURE 2:**
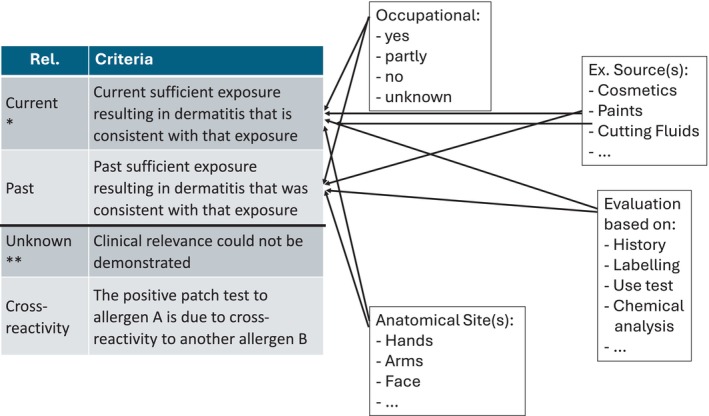
Pragmatic approach to scoring clinical relevance of a positive patch test to an allergen in an individual patient combining different EU and US/UK proposals, expandable by addition of further details. Rel., clinical relevance; Ex., exposure. *Current clinical relevance ideally also includes a follow‐up of 3–4 months to evaluate amelioration of the dermatitis, preferably complete or at least partial in case of multifactorial disease (e.g., hand dermatitis); additionally, and further strengthening the relationship, the dermatitis might also relapse upon re‐exposure to the allergen **‘Unknown’ relevance is preferred to ‘no’ relevance, as there may be several reasons why relevance could not be established (Section 7.2).

No commonly accepted relevance scoring system exists, but different proposals have been suggested in Europe, US and UK (Figure 2) [135, 136, 137, 138, 139, 140, 141]. Pragmatically, a positive patch test reaction can be of current and/or past relevance, or unknown relevance. The designations ‘current’ and ‘past’ relevance can be complemented with a further qualifier addressing the likelihood of the relevance statement, for example, like the relevance scoring used in the US and some UK centres (definite, probable and possible, respectively) [139, 140, 141]. A positive patch test may also represent a cross‐reaction to another sensitizer that may be of clinical relevance, for example, a positive patch test to *p*‐phenylenediamine (PPD) may actually indicate relevant exposure and sensitization to diaminodiphenylmethane (MDA). If such a substance (MDA) ‘cross‐reacts’ with a diagnosed allergen (PPD), previous exposure and sensitization to the cross‐reacting substance (PPD) is not necessary [142].It is important to understand that an irritant reaction observed in patch testing does not establish a diagnosis of ICD to the tested substance.

Although European and US relevance grading systems are useful for the individual patient, demonstrating a good connection between the allergen and type of relevance (past/present/unknown), there are sometimes difficulties, especially in epidemiological studies, to connect the allergen(s), relevance and (type and location of) dermatitis investigated [64]. An improved, easy‐to‐use and standardized method of data collection has been proposed, especially useful within the frame of (online) clinical documentation/software (e.g., when testing and reporting about consecutively tested patients) that more accurately links (positive patch tests to) allergens to exposure, clinical disease types and patient history, and thus better represents clinical relevance, enabling a more precise analysis of causation [143]. Briefly, the approach combines information already collected in traditional documentation, but combines this in a way to form a type of combination of data reflecting timing (current vs. past), diagnosis of the dermatosis (ACD vs. ICD vs. other), occupational causation, anatomical site(s), allergen, culprit sources and the source of information on which a judgement is based, that is, patient history, patch test with the product, use test with the product, ingredient labelling, SDSs, workplace visit or chemical analysis; several combinations can be documented even for one allergen putatively causing or having caused ACD by different exposures, affecting different anatomical sites at different times. The ‘COADEX system’ proposed in 2004 [112], partially based on an (updated) British guideline [68, 144], for categorizing clinical relevance may represent a further alternative for relevance assessment [145]. It should be noted that the strength of a patch test reaction does not necessarily correlate with clinical relevance.

**Conclusion**: Current clinical relevance implies that the contact allergy can completely or partially explain dermatitis leading to present consultation. To be relevant, exposure to an allergen needs to be sufficient and consistent with the patient's history and the clinical disease, the latter then diagnosed as allergic contact dermatitis.

- It is recommended that the appropriately qualified physician always assesses whether a morphologically positive patch test (contact allergy) is of current, past or unknown relevance, or is attributable to cross‐reactivity (100%).
- It is recommended that for relevance assessment, exposures at home, at work, and during recreational activities are addressed (100%).

### 7.2 Current Clinical Relevance

Exposure assessment is mostly guided by history [24]. It is therefore important to scrutinize the patient's history systematically. It can be helpful to ask about rashes resulting from the use of specific product types, for example perfumes, creams, gloves, shoes, tools and jewellery, and including consort dermatitis [146], depending on the anatomical site of dermatitis and the allergy under investigation, perhaps in a standardized way [143]. Nevertheless, patient recall may be of limited value in establishing the clinical relevance of positive patch test reactions, for example, to fragrances [147, 148].

If a particular product is suspected on the basis of the history, the presence of the diagnosed sensitizer must be demonstrated, for example, by referring to the ingredient labelling in case of cosmetics (INCI system). In Europe, the labels of household detergents list preservatives and fragrances according to the requirements of cosmetic products. Labelling on medicinal products is also compulsory, and follows the INN system, whereas (topical) medical devices are, unfortunately, rarely labelled [149], which is a deficiency in current legislation [150].

For solid product types, such as shoes, gloves, textiles and furniture, it is usually impossible to obtain information about their composition; both literature reports and textbooks can be consulted for information.

For occupational products, also the SDS should be checked, and workplace visits may be required. Additionally, also for consumer products, contact with manufacturers and/or chemical analyses, if available, may be necessary and useful. Particularly, as ingredients of these products are usually not labelled if their concentrations are below the threshold level for legally required labelling or regulations for specific products (e.g., biocidal products) allow for labelling of only active ingredients and omitting others [151].

For certain allergens, such as nickel, cobalt, chromium and formaldehyde, spot tests exist, which are quick and easy ways to assess exposures. The nickel spot test is the best validated, and has high specificity (97.5%) and moderate sensitivity (59.3%) in detecting a level of nickel ion release that may cause dermatitis [152]. In cases of suspected (occupational) exposure, the nickel spot test can also be used directly on the hands [153]. The cobalt test is based on similar principles, but is more difficult to read, and there is less experience with the test [154]. Important new sources of exposure to cobalt have been identified by use of the cobalt spot test [155, 156, 157]. The formaldehyde (chromotropic acid) spot test requires laboratory facilities, but can detect small levels of formaldehyde, which have been shown to be of clinical relevance in those sensitized [158]; ready‐to‐use formaldehyde spot tests have been insufficiently validated [159]. The diphenylcarbazide test can detect chromium (VI) [160], yet is not commercially available.

**Conclusion**: Exposure assessment to verify the relevance of a positive patch test reaction to an allergen may include.

- a detailed patient history, verification of INCI ingredient labelling of cosmetic products and household detergents,
- checking International non‐proprietary Names (INN) labelling of medicines,
- scrutiny of Safety Data Sheets of occupational products.
- In case of solid items, medical devices and occupational products composition may be more difficult to retrieve, and contact with manufacturers or chemical analyses may be required.
- In case of contact allergy to nickel, cobalt or formaldehyde, use of spot tests to identify sources of exposure at the workplace and at home.

Definite relevance of natural substances may sometimes be difficult to elucidate. Cross‐reacting substances should also be looked for in the environment, as these may be (equally or more) relevant.

A special challenge occurs if the positive patch test reaction is to a mixture that is used for screening of contact allergy to a group of substances, such as fragrance or rubber mixes, and especially if it concerns natural mixtures such as *Myroxylon pereirae* resin (balsam of Peru) or colophonium. In a mix of chemicals or natural extracts (e.g., the fragrance or rubber chemical mixes) the different constituents can be patch tested separately, but when it concerns a natural mixture, it may not always be possible to pinpoint a particular sensitizer. The relationship between a dermatitis and use of a product containing such related substances, supported by knowledge from literature and textbooks, is often the only way to state anything on (possible) relevance.

The possibility of cross‐reactivity, see above, should also be kept in mind. If the clinician looks for the substance that has caused the positive patch test reaction, and this is not present in the environment, the wrong conclusion may be drawn that the allergy is not relevant. Alternatively, if the substance is (also) present, the true culprit exposure could still be overlooked. If, on the other hand, a patient reacts to a given hapten, and also to one or a few known cross‐reactors, relevance may be ‘transferrable’ to these cross‐reactors [161], yet clinical relevance may also remain unclear, and thus unknown [162]. Indeed, a cross‐reactor can be relevant on its own, but might also just indicate patch‐test cross‐reactivity with unknown relevance [163].

Further instruments in the assessment of clinical relevance [134] include patch testing of products ‘as is’ or appropriately diluted, patch testing with extracts, ROATs [106], or modifications thereof (e.g., GRAT, glove repeated application tests) [108], and use tests, if this has not been done in the original diagnostic work‐up of the patient already.

A positive patch test reaction to a suspected product supplied by the patient in which the sensitizer is an ingredient and to which the patient is exposed usually means that the contact allergy is relevant [134]. In case a patient shows a positive reaction to a product ‘as is’ or when tested as an extract, and also reacts to a defined sensitizer, it is still necessary to demonstrate the actual presence of that sensitizer in the product, even more so when a potentially new skin sensitizer is concerned.

Despite careful exposure assessment, current clinical relevance may remain difficult to judge, in every stage of the investigation [82, 164], owing to multiple (non‐exhaustive) reasons:

- suspected products may have been discarded by the patient, can be mislabelled, insufficiently labelled or unlabelled, compositions may have changed, or hidden sensitizers may be present (e.g., formaldehyde) [165]. Additionally, some sources of the substance in question may not have been traced;
- a negative patch test result with a product, and even an extract, still does not exclude (current) clinical relevance. However, even a negative ROAT or use test result does still not entirely exclude relevance;
- contrary to medicines, medical devices (e.g., various implants, but also diabetes sensors and pumps) constitute a particular problem as these are rarely labelled and their compositions may vary without notice [149];
- information from manufacturers may be unavailable or unreliable;
- SDS of occupational products may be incomplete [166], or regulation‐based high labelling thresholds for some ingredients prevent disclosure of, for exampple, biocidal allergens
- a substance may be present in the general environment so ubiquitously that the significance of the exposures cannot be clarified from the history;
- the patient has never (or not yet) developed dermatitis caused by the substances, as the patient has not been exposed to sufficient amounts after initial clinical or previous patch test sensitization;
- contact has occurred only with a cross‐reacting substance, which may have a different usage;
- the patient's memory may be unreliable [148], or lack of knowledge exists on the part of the clinician, or he/she is unable to ask the proper questions.

It remains equally difficult to establish the relevance of contact allergies in case of implant‐related problems (where mere sensitization may have occurred, and other explanations may co‐exist or prevail) [167], or in cases where polysensitization occurred to several allergens [168], or when non‐cutaneous symptoms dominate the clinical picture. If relevance for a given allergen can eventually not be established, despite all these considerations, it is recorded as a patch test reaction of ‘unknown relevance’ [169].

### 7.3 Unknown Relevance

The term ‘unknown relevance’ is preferred to ‘no clinical relevance’, and should be cautiously used, as there may be several reasons for not having detected the relevance. Importantly, a given sensitizer associated with ‘unknown relevance’ does not imply that the morphologically positive patch test reaction obtained with it can be automatically labelled as false‐positive, as additional scrutiny may have been required to establish relevance, as often, for example, in the case sulphites [2]. Nevertheless, if a given hapten consistently, in a majority of cases, results in morphologically positive reactions, yet without clear relevance, it may indicate a knowledge gap concerning potential hidden sources of the allergen, such as for methyldibromo glutaronitrile [170], or a problematic composition of the patch test material (e.g., benzisothiazolinone), the latter sometimes suspected when many weak (+) patch test reactions without obvious relevance occur in consecutive patients [171].

- It is recommended, in the case of unknown relevance of a positive patch test reaction, to re‐evaluate the history and exposures, to repeat the clinical examination, and to consider additional tests (e.g., ROATs, use tests, spot tests, chemical analyses and worksite visits) where indicated (100%).
- It is not recommended to regard a morphologically positive patch test, yet with ‘unknown relevance’, as a false‐positive test result (100%).

### 7.4 Past Relevance

Past relevance reflects a past episode of contact dermatitis caused by exposure to the allergen, for example previous contact dermatitis caused by an earring in a person with a positive patch test reaction to nickel. Past relevance is usually based mainly on the patient's history. On a population level, relevance for a given sensitizer may shift from current to past relevance and, regarding culprit sources, from cosmetic to non‐cosmetic products, often reflecting a positive impact of regulatory interventions [172].

### 7.5 Interpretation of Doubtful Patch Test Reactions

An erythematous (?+; doubtful) patch test reaction means that the morphology is not clear‐cut allergic or irritant. Although often not assessed for relevance, some doubtful reactions may be truly allergic, and could impact patient care [173]. Ideally, further investigations thus need to be performed. Several scenarios can be considered:

- a doubtful patch test reaction may be attributable to cross‐reactivity to another substance, which is the primary (and often stronger) sensitizer. Consideration should thus be given to the pattern of reactions. If reactions to some chemicals from the same family are doubtful, and others are (strong) positive (e.g., reactions to several formaldehyde releasers, rubber chemicals or fragrance substances) this may be a sign of the same contact allergy. Further testing is not always required, and relevance assessment can be conducted, for the individual sensitizer, and/or (pragmatically) also regarding group allergy (e.g., rubber allergens [dithiocarbamates/thiurams]);
- the patch test concentration may be marginally irritant, and the doubtful reaction may just be a sign of mere skin irritation. Repeat patch testing (yielding the same result) and/or serial dilution patch testing with the application of both higher and lower test concentrations, resulting in a sharp cut‐off with higher dilutions, may help in diagnosing the reaction as irritant. No relevance assessment is required in this case;
- As regards a true allergic reaction, the patch test concentration used may have been too low. If retested, the reactivity may be enhanced (‘boosted’) and produce a clear positive reaction. Alternatively, when the test concentration is increased, a positive patch test reaction may also develop, which can be of current clinical relevance. If, for instance, formaldehyde is tested only 1% aq., no or a doubtful reactions may occur, and truly positive reactions are missed, yet picked up by testing 2% aq., the latter also shown to be clinically relevant by use tests with formaldehyde‐containing creams [158]. Evidently, doubtful allergic reactions regularly occur to lower test concentrations of an allergen that is clearly positive at higher concentrations in serial dilution testing;
- Corticosteroids may lead to erythema only, presumably owing to a partial suppression of the patch test elicitation reaction owing to their pharmacological effect. Further, the aminoglycoside antibiotic neomycin (sulphate) is known to cause erythematous allergic reactions [174];
- Patch testing with suspected culprits of eyelid or mucosal contact allergy may yield doubtful reactions, despite clinical relevance, as the standard test site, the back, is considerably thicker than the eyelid or mucosal skin, leading to reduced penetration of contact allergens

It is recommended in case of an isolated doubtful reaction, suspected to be allergic, to re‐test (expectedly with enhanced reactivity in case of contact allergy) and/or perform serial dilution testing to clarify the true nature of the reaction (95.2%).

### 7.6 Final Diagnosis

If current clinical relevance is found in a person with established contact allergy, the diagnosis of ACD can be made. To unleash its full potential for being informative, an ACD diagnosis (or several, if indicated) should link (current or past) dermatitis of the anatomical site affected, possibly with information on recurrence, chronicity or duration, with the respective contact allergy/allergies diagnosed. Independently, re‐assessment of clinical relevance can be helpful in terms of assessing clearance of dermatitis after 3–4 months of proper allergen avoidance [140]. In the case of unknown relevance, the person is sensitized, that is, has a contact allergy, but the criteria for the diagnosis of ACD have not currently been met. However, the person is at risk of developing ACD in the future if sufficiently exposed to the allergen (possible ‘future relevance’). Hence, contact allergy with unknown relevance must also be mentioned in the list of diagnoses, and counselling of the patient should include the respective substance(s). In some cases, exposure to a contact allergen may explain the dermatitis entirely, but skin conditions with a multifactorial background frequently occur, for instance in hand dermatitis. Besides exposure to one established contact allergen, also other allergens and irritants may be involved, and constitutional factors such as atopy can also be of importance. All these factors should be appropriately reflected in the diagnoses finally made, acknowledging it may be difficult to assess the relative significance of these various factors at a given time [134], again pointing to the usefulness of a follow‐up re‐evaluation.

## 8 Influence of Individual and Exogenous Factors

Historical data were reviewed by reference to relevant chapters in Rook's Textbook of Dermatology [175] and the ‘Contact Dermatitis’ textbook [5]. This was supplemented by searching PubMed with relevant search terms, and a hand search of the indices of the journals ‘Contact Dermatitis’ and ‘Dermatitis’ from 2018.

When performing patch testing, it is important, among other factors, to consider the responsiveness of the patient. Individual factors such as age and exogenous factors (e.g., drugs, immunosuppressive diseases, UV radiation) may theoretically weaken the patch test response, resulting in false‐negative reactions, whereas other factors may increase the response, such as active or widespread dermatitis, resulting in false‐positive or irritant reactions. Much evidence within this area is based on clinical experience, and limited controlled data are available.

### 8.1 Medication

There is little information in the literature about the effects of immunosuppressive/immunomodulating agents on allergic patch test reactions. In practice, it may be difficult or impossible for patients to stop using their immunosuppressive/immunomodulating drugs, for example corticosteroids, azathioprine, cyclosporine, biologics and Janus kinase (JAK)‐inhibitors. In such circumstances, patch testing can be undertaken, but the clinician must be aware that false‐negative reactions can to occur.

However, positive reactions may still occur in patients undergoing treatment for psoriasis not only treated with the aforementioned immunosuppressive traditional drugs but also with methotrexate and anti‐TNFα drugs [176, 177]. Similarly, patients with atopic dermatitis treated with immunosuppressive drugs (e.g., low‐dose prednisolone, cyclosporine and methotrexate) [177] and immunomodulating biologics like dupilumab [178], can also show positive patch test reactions. Regarding dupilumab, divergent results have been published and positive patch test reactions may disappear from 3.4% [178], to 24% [179] and even up to 71% of patients [180]. Biologics for atopic dermatitis may alter the patch test result profiles of specific allergens [181, 182], although it has also been reported that one third of positive patch test reactions was confirmed independently from the allergen, depending mostly on the strength of the patch test reaction [180]. Regarding the effect of JAK‐inhibitors on patch testing results, only few data on upadacitinib are available, showing false‐negative reactions to selected allergens [183, 184]. High‐dose or systemic corticosteroids (especially > 20 mg prednisone equivalent/day) can suppress or eliminate patch test reactions [185].

To minimize the potential influence of systemic treatments on the patch test, it is recommended to stop them about five half‐lives before the test as rule of thumb. Based on a number of studies [186, 187, 188, 189], antihistamines seem to have potential for causing a decrease of reactions. Some experts consider this not to be relevant [181]. For alitretinoin, there are no data in the literature. Low/medium potency topical corticosteroids applied to the test area should be stopped 7 days and high potency 14 days prior to the patch test [181]. Also topical calcineurin and JAK inhibitors may have an inhibitory effect on patch test response similar to corticosteroids [190].

- It is recommended in case of a strong suspicion of ACD to patch test also patients under immunosuppressive therapy (100%).
- It is recommended to patch test before starting a systemic therapy, if possible, because patch testing under immunosuppressive therapy may result in false‐negative reactions (100%).
- It is recommended to avoid topical corticosteroids and calcineurin inhibitors on the test area for 7–14 days (depending on potency and cumulative dose) preceding the patch test (100%).

### 8.2 Immunosuppressive Diseases

Some diseases characterized by altered immune response such as HIV/AIDS (unless at an early stage or well‐managed), hematologic malignancies, organ transplant status, autoimmune conditions under immunosuppressive therapy, primary immunodeficiencies and advanced cancers may impair T‐cell–mediated immune responses, potentially leading to false‐negative or weaker patch test reactions. Besides cancer itself, also therapies for the cancer could influence patch test results [191].

It is recommended to proceed with patch testing, if allergic contact dermatitis is suspected in patients with immunosuppressive diseases, but to keep in mind that false‐negative reactions may occur and, if possible, to repeat patch testing at a later stage (100%).

### 8.3 Atopic Dermatitis and Concomitant Active Eczematous Disease

In most studies, the frequency of positive patch test reactions in atopic dermatitis subjects is similar to that observed in patients without and therefore, patch testing is recommended in atopic dermatitis patients when ACD is suspected [63]. A recent meta‐analysis did not reveal an overall difference between patients with versus without atopic dermatitis with regard to contact allergy prevalence [192]. Nevertheless, the interpretation of patch test results in atopic patients may be difficult owing to their generally hyperreactive skin with a risk of false‐positive (irritant) reactions; re‐testing or verification tests like ROAT can be decisive for arriving at a proper diagnosis. The association between filaggrin mutations, leading to impaired epidermal barrier function and contact allergy, is controversial: some studies documented a slightly increased risk [193, 194], while other reported no association [195].

It is recommended to patch test patients with atopic dermatitis (AD) when allergic contact dermatitis is suspected, for example, with lack of improvement of apparent AD under adequate treatment, ideally before systemic therapy (95.2%).

### 8.4 UV Radiation/Sun Exposure

UV radiation is a well‐known suppressor of type IV allergic reactions, with the degree of suppression depending on UV cumulative dose, wavelength and other factors. Although this seems not to be the case for UVA [196, 197] it is historically known that psoralen combined with UVA can cause a reduction in patch test reactions [198], as well as a depletion of Langerhans cells in mice [199]. UV irradiation results in a reduction in epidermal Langerhans cell numbers also in humans [200]. According to studies in mice, such depletion returned to normal after 6 weeks [201], while in human volunteers full recovery was observed within 4–5 weeks [202]. Avoidance of around 4 weeks after intense sun exposure and after UV‐therapy is recommended, while 6 weeks suggested by some guidelines do not appear entirely necessary [203]. This notion is corroborated by the recent observation that UV radiation induces epidermal recruitment of dendritic cells within days post irradiation that probably immunologically compensate for the depletion of Langerhans cells [204].

### 8.5 Temperature and Ambient Air Humidity

Adhesion of patches may be compromised in hot and humid weather conditions and thereby affect patch test results. Moreover, ambient temperature and humidity may influence reactivity to contact allergens, for example, by impacting the skin barrier function. Low ambient temperature and absolute humidity, respectively, may compromise the epidermal skin barrier [205], and thereby increase the frequency of irritant or doubtful reactions to certain allergens, such as paraben mix and methylisothiazolinone; however, the increase of weak positive reactions associated with low temperature and humidity seen with, inter alia, fragrance mix, methyldibromo glutaronitrile, *p*‐phenylenediamine and particularly formaldehyde (tested in water) is more concerning, as some share of these ‘+’ reactions may be false‐positive [206, 207]. However, the majority of (baseline series) contact allergens was found largely unaffected by meteorological conditions [207]. Conversely, high temperature and absolute humidity can increase skin hydration and blood flow, potentially leading to enhanced allergen absorption.

It is recommended to delay patch testing for around 4 weeks after intense sun exposure of the area of application of the patch test, and 4 weeks after UV‐therapy (95.8%).

## 9 Special Groups

### 9.1 Children

The literature search was performed with PubMed using the following keywords in various combinations: ‘allergic contact dermatitis’, ‘children’, ‘baseline series’ and ‘patch testing’. Recent articles focusing on epidemiological data and adverse effects associated with an allergen, as well as those discussing concentrations of allergens or abbreviated baseline series were selected.

#### 9.1.1 Introduction

ACD in children is frequent across different geographical regions but it has been under‐recognized for a long time. Over the past decade, significant progress has been made in understanding the allergens responsible for contact allergy in this age group. All children, whether atopic or not, may become sensitized to environmental chemicals such as topical pharmaceuticals, cosmetic products and those used by their care‐givers (dermatitis by proxy), jewellery, toys or any other material that comes into prolonged contact with the skin [208, 209, 210, 211, 212, 213]. They may also be sensitized by allergens often regarded as occupational allergens by recreational exposure, such as epoxy resins [214]. The spectrum of contact allergens in children and adolescents is like that of adults; however, compounds such as colophonium, and limonene and linalool hydroperoxides have recently been found to be more common in the younger age groups, which underscores the burden of fragrance sensitization [213]. Patch testing in children is considered safe, and is recommended when ACD is suspected or needs to be ruled out, as in adults.

#### 9.1.2 Technique and Allergens

Patch testing may present practical challenges, particularly in very young children. The technique is the same as in adults [215]. However, certain factors must be considered, such as the smaller test area on the back and the greater mobility of younger children, requiring the use of stronger adhesive tapes. Due to space limitations, it may sometimes be impossible to test the whole baseline series, and a selection of contact allergens is therefore required. A paediatric baseline series has been previously proposed [208], and a proposal for a paediatric EBS has recently been published on behalf of the EBS working party of the ESCD [8]. Children with and without atopic dermatitis may be sensitized by different allergens, which prompted an adapted baseline series for the latter population in Portugal [213].

On the other hand, patch testing with the products that children are exposed to, such as topical products, medical devices, antiseptics, cosmetic products including gel nails, or toys and, if possible, also with their potential ingredients, is crucial (see Section 6) [30]. In very young children, in particular, products considered as possible cause of ACD may sometimes be the only materials to be tested.

**Conclusion**: The indications for patch testing in children are the same as in adults. Patch testing is safe, and the use of a paediatric baseline series is recommended.

### 9.2 Occupational Contact Dermatitis

A search for publications since 2016 was performed using PubMed with the following search terms: ‘Patch Tests’[Majr] AND (occupation* OR work*) with 185 results; (‘Patch Tests’[MeSH] AND (occupation* OR work*)), 893 results; (‘Patch Tests’[MeSH] AND (‘occupational exposure’[mesh])), 78 results; further, four articles quoting this guideline. In Embase, the search (‘patch test’/exp. OR ‘patch test’) AND (‘occupation’/exp. OR ‘occupation’) AND [2015–2025]/py has been used. In addition, chapters from the 3rd edition of ‘Kanerva's Occupational Dermatology’ handbook (2020) [6] were considered.

Patients presenting with possibly work‐related contact dermatitis require a number of special considerations, as outlined in the following section. These are in line with minimum standards on prevention, diagnosis and treatment of occupational and work‐related skin diseases in Europe, which have been published by the COST Action StanDerm [216].

#### 9.2.1 Definition

There is no consensus on the definition of occupational contact dermatitis. It was suggested recently to prefer the term ‘work‐related’ contact dermatitis [216, 217]. It can include any type of contact dermatitis caused by occupational exposure: either exclusively or predominantly or partial. ‘Work‐related’ is independent of legal definitions, which vary between (EU) countries. As some contact allergens are ubiquitous, contact sensitizations acquired at the work place may well give rise to symptoms in patients' private life, and vice versa.

#### 9.2.2 Patient History

In addition to a standard history, present and previous employments, occupational exposures, work tasks, leisure activities and other relevant aspects need to be documented in detail (and may be required later for medico‐legal purposes). Some more common occupations may be familiar to physicians, depending on their experience. Nevertheless, checklists may help to cover all relevant aspects and, at the same time, assist documentation [6]. Other occupations may require consultation of textbooks [5, 6] or information resources on the internet (see Section 13) to appreciate the array of relevant exposures. It has been pointed out that sketches or photographs (enabled by mobile phones) provided by the patient can be very helpful in identifying an exposure‐related problem. Visits to patients' workplaces often provide crucial information concerning hazardous exposures, which may not be obvious to patients, workers or employers.

#### 9.2.3 Exposure Analysis

After the collection of basic information from the patient's history, it is often necessary to proceed to in‐depth analyses of occupational exposures of the patient. Exposure analysis includes two levels:

1. Collection of products and materials handled by the patient, along with information on their ingredients, for example in terms of SDSs. However, even though a particular substance is not mentioned, it may be present, as only classified skin sensitizers have to be mentioned if they are present above a certain concentration limit in chemical products (mixtures) [218, 219, 220]. Thus, clinically important and frequent allergens may not be listed on SDSs even with formally correct, but factually incomplete, declarations [160]. It is therefore advisable to contact the manufacturer or supplier of the product(s) under suspicion to obtain a full list of ingredients.
  Furthermore, SDSs are not, by EU legislation, required for solid materials including textile, leather, rubber or polymerized materials, nor for personal protective equipment (gloves, clothes, goggles etc.) and labelling is clearly incomplete. Labelling can be incorrect and hampers prevention of ACD. Chemical analysis found rubber accelerators in gloves labelled as ‘accelerator free’ [221, 222].
2. Actual chemical analysis with suitable laboratory tests of working materials deemed to be possibly relevant [160]. Some spot tests are useful to screen the (working) environment for the presence of these allergens (see Section 7).

Ideally, exposure analysis should also include assessment of how much of the allergen is deposited onto the skin [153, 160]. Such information may be used when the occupational relevance of patch test reactions is assessed and exposure reduction is planned. Only a few methods for the assessment of skin exposure to common allergens, such as some metals, hair dyes and epoxy and acrylate resins, are currently available [160]. The simplest detection method is that for nickel, where the dimethylglyoxime test on the skin, which is easily applied in the clinic or workplace, may be used for qualitative assessment of nickel exposure [153].

Regarding the application of special patch test series, such as hairdresser series and cutting fluid series, a case‐by‐case extension of these requires sufficient knowledge of the patient's exposure; referral to specialized institutions is advised. A missed allergen or allergens, besides other causes, must be suspected in cases of persisting skin problems.

#### 9.2.4 Patch Testing With Work Materials

Patch testing of standardized patch test preparations and material from the patients' workplaces is the gold standard in the diagnosis of occupational contact dermatitis. The diagnosis of occupational ACD can be hampered in some countries by a limited number of authorized commercial test allergens available on the market [22].

Hence, patch tests with work material are an even more important starting point to diagnose occupational ACD, and also for detecting new allergens and new sources of exposure [223]. It was reported that testing with workplace materials and in‐house test substances yielded almost 20% additional occupational ACD diagnoses [116]. These cases would have been missed with only commercially available patch test substances. It should be noted that new substances, chemically similar to known skin sensitizers, are introduced by industry. They may not yet have been classified as skin sensitizers and information about their use is thus often lacking. Examples are various isothiazolinones and polymers [220]. The recommendations in the Section 6 should be followed.

#### 9.2.5 Relevance Assessment and Final Diagnosis

Final evaluation with assessment of the clinical relevance of the patch test result is described in detail in Section 7. Occupational exposures to chemicals with which the dermatologist has little experience makes this a particularly difficult task. The assessment may have a direct impact on the prognosis of the patient's dermatitis and future work career, on medico‐legal decisions, including compensation or re‐training and on preventive measures in the workplace.

Regarding occupational relevance, the following two aspects need to be considered:

- The association between the onset and the course of dermatitis (improvement or healing when away from work; relapse after return to work) and the affected anatomical site (hand, face or other sites directly or indirectly exposed by airborne dust or liquid aerosol, gas, drips or spills or other contamination).
- Exposure to work materials containing the allergen.

Allergens may be relevant both occupationally and in a non‐occupational context, and the relative contributions of the two exposure arenas need to be estimated to determine if the exposure to the allergen has been more pronounced at work or at leisure time. A statement on relevance should also include a reference to time, that is, whether relevance is current or past.

Finally, one diagnosis or several diagnoses need to be made, each with a statement concerning the role of occupational exposure, which can be the sole, the predominant or a contributory cause, or not be a cause at all. Ideally, each diagnosis should give information on the affected anatomical site, the causative exposure/work material, and the actual allergen(s) (if ACD or contact urticaria) or irritant(s) (if ICD) involved and, moreover, whether any pre‐existing disease or disposition (mainly atopic dermatitis) or exogenous co‐factors such as occlusion and friction are involved. It should be noted that the WHO ICD11 allows for such very detailed documentation, including occupational causation.

It is recommended to systematically evaluate patients with work‐related dermatitis addressing diagnoses, the affected site, the offending work material and the causative allergen or irritant for optimal patient counselling and exposure reduction (100%).

### 9.3 Testing in Drug Reactions

Diagnostic patch testing is an investigation undertaken on patients with a history of dermatitis (eczema) to determine whether they have a contact allergy and then evaluate its relation (if any) to their dermatitis (see Section 7). Patch testing should be performed in all patients in whom contact allergy is suspected or needs to be excluded, regardless of, for example, age (see Section 9.1) or anatomical site of dermatitis. This also includes (i) non‐eczematous conditions that may represent a contact allergic reaction, such as erythema multiforme‐like, lichenoid and psoriasis‐like lesions, as well as granulomatous and lymphomatoid reactions [224], (ii) worsening of pre‐existing dermatitis, such as stasis, atopic and seborrheic dermatitis, and nummular eczema, (iii) certain drug eruptions (see section ‘Patch testing in drug eruptions’) [129], (iv) mucous membrane reactions, for example, conjunctivitis, stomatitis [225] or vulvitis [226], balanoposthitis, perianal dermatitis, (v) clinically suspected delayed‐type hypersensitivity reactions to dental or orthopaedic implants [227], and stents [228].

### 9.4 Patch Testing in Eruptions Related to Drugs

#### 9.4.1 Indications

Although less standardized in this context, patch testing with drugs may also be indicated in the investigation of delayed cutaneous adverse drug reactions (CADRs) (beginning > 6 h after drug intake), namely in maculopapular exanthema (MPE), drug reaction with eosinophilia and systemic symptoms (DRESS), acute generalized exanthematous pustulosis (AGEP), fixed drug eruption, (FDE), systemic‐drug‐related intertriginous and flexural exanthema (SDRIFE), Stevens‐Johnson syndrome and toxic epidermal necrolysis (SJS/TEN) [229, 230, 231, 232]. In Europe, a consensus recommends to initially perform patch tests and subsequently, if these are negative, and if it concerns a non‐severe CADR, to continue with prick tests and intradermal tests (IDTs) with delayed readings [232]. Drug testing is usually undertaken in specialized departments.

Moreover, in cases of skin contact with drugs (e.g., during manufacture or handling) resulting in suspected ACD, patch testing is also indicated, and the technical procedure is the same.

#### 9.4.2 Method

Optimally, patch testing should be performed at least 4–6 weeks after complete resolution of the CADR and, preferably, within 1 year after the disappearance of the drug eruption. In patients with DRESS, patch tests are optimally performed at least 6 months after the disappearance of the CADR and in the absence of high virus replication (EBV, HSV, etc.) as co‐factor. All possible culprit drugs should be tested, that is, all drugs taken within the relevant chronological period for each type of CADR.

The approach and technique for performing patch testing in CADR is the same as that for the investigation of ACD. There is only the exception of FDE, where patches should be applied both to previously affected and to healthy unaffected skin. A recent study has shown that a sequential allergy work‐up with successively applying in situ patch tests, in situ ROAT over a 7‐day period, and finally drug challenge in case of negative skin tests was a reliable and safe method for diagnosing the cause of FDE. In situ tests exhibited a sensitivity of over 50% [233].

For all other CADRs, patch test readings should be performed as for ACD and also read twice between day 2 and 5. A later reading at D7 is strongly recommended especially for corticosteroids [229, 230, 231]. Apart from erythema and papules, and possibly vesicles, different clinical and histopathological reaction patterns (pustular, lymphomatoid or bullous) may occur in positive PT to systemic drugs. They mostly resemble the acute CADR, both clinically and on histopathology [230], but do not depend on the culprit drug [234, 235, 236, 237, 238].

#### 9.4.3 Allergens for Patch Testing in Patients With CADRs

Few drug allergens are commercially available for patch testing, such as some antibiotics, NSAIDs and anticonvulsants, usually at 10% pet. Therefore, in most cases, patch test material has to be prepared in‐house from the drugs used by the patients, adhering to pharmaceutical GMP standards. For preparation of this patch test material, the powder from intravenous preparations or from capsules is preferred over tablets. This powder needs to be finely grinded and incorporated in pet., whenever possible, to achieve a final 10% (w/w) dilution of the active principle. When the concentration of the active drug is too low in the patient's drug, the powder as a whole should be diluted to 30% pet [130, 229, 230, 231, 239]. Positive patch test results obtained with these in‐house preparations should be validated with controls, if possible, as some drugs or their excipients may have irritant properties, as shown, for instance, for colchicine, captopril and desloratadine [230, 240]. For commercially available drug allergens, no further controls are needed [239].

#### 9.4.4 Sensitivity and Specificity of Patch Testing in Patients With CADRs

Patch test specificity is usually high, with drug‐specific T cells being isolated from positive patch test reactions and positive patch tests showing the histopathology of the acute CADR [241]. Patch test sensitivity in patients with CADRs is lower than in patients with ACD (30%–70%), and depends on the culprit drug and the clinical pattern of CADRs [230, 242]. Some drugs are frequently patch test positive, such as antiepileptics, 𝛽‐lactams, iodinated contrast media and pristinamycin [229, 230, 231, 243, 244].By contrast, in other drugs, such clindamycin, a low percentage of reactivity is expected (20%–30%) [245], probably reflecting reduced absorption, the role of metabolites, or the need for concomitant factors for induction of the CADR. Allopurinol and sulfa drugs such as salazopyrine or cotrimoxazole, usually give negative patch test results [230, 231, 246]. Patch tests in patients with MPE, DRESS, AGEP, FDE, SDRIFE give more frequently positive results than in patients with SJS/TEN [230, 231, 240, 247]. These latter CADR show a low sensitivity of < 30% [230, 231, 232, 247].

Prick and intradermal tests, with immediate and late readings, can have additional value in the assessment of patients with CADRs, but they are beyond the scope of this guideline [230, 231, 232]. Scratch patch tests are sometimes useful but some comparative studies with patch testing are needed [101, 248]. Patch testing is a safe procedure, even in patients with severe CADRs, apart from exceptional cases of minor reactivation of the CADR [231]. Information on non‐irritating concentrations of active ingredients in drug patch tests has been compiled some years ago [249].

**Conclusion**: A positive patch test result can help to confirm a possible culprit drug, therefore avoiding oral provocation. A negative patch test result, on the other hand, cannot exclude the contribution of a possible culprit drug, determined on clinical grounds.

It is recommended to consider patch testing in patients with delayed cutaneous adverse drug reactions (CADRs), although it has variable sensitivity (95.2%).

## 10 Potential Adverse Effects of Patch Testing

Using the search engines: PubMed and Web of Science, the following key words were used for retrieving references: Patch test/testing and (successively, one of the following terms) active sensitization; adhesive; adverse effect; adverse event; anaphylactoid; angry‐back; burning; complications; depigmentation; discoloration; epicutaneous allergy test; excited skin; flare; flare‐up; granuloma; granulomatous; hazard; hyperpigmentation; immediate reaction; irritant; irritation; itching; late reaction; leukoderma; lichenoid; long‐lasting; long‐standing; lymphomatoid; pain; patch; patches; persistent; persisting; plaster; procedure; prolonged; pruritus; pseudolymphoma; sarcoid; sensitization; side effects; subjective complaints; subjective symptoms; tape reaction; testing; ulcer; unexpected; urticaria; unwanted. In addition, relevant textbooks were screened [2, 3, 5].

The following section on adverse effects of patch testing refers to tests performed appropriately and correctly, in accordance with these guidelines, with commercially available materials, patch test materials prepared in line with reference textbooks such as [32], or patient's own materials after careful safety/toxicological evaluation. Patch testing is generally a safe diagnostic procedure; adverse events, usually (extremely) rare, are discussed below.

### 10.1 Unexpected Irritant Reactions

Irritant reactions are mostly observed with non‐standardized test allergens or non‐appropriately diluted or pH‐adjusted materials brought in by the patient, or by the adhesive parts of patch test plasters or chambers.

### 10.2 Active Sensitization

Sensitization by patch testing is a very rare complication for standardized allergens. Active sensitization is suspected if a very late positive patch test reaction, generally beyond 10–14 days, is observed, followed by an earlier positive test within 7 days upon repeated patch testing [64]. In practice, it may be difficult to differentiate between induction of sensitization from allergen exposure from the patch test and a delayed patch test elicitation reaction [250]. To confirm the diagnosis of active sensitization, repeat patch testing must be performed. A positive reaction, with ‘normal’ latency of elicitation (one to a few days), supports patch test sensitization, particularly if there is a positive reaction to the test preparation diluted 10–100 times [251]. However, boosting of a pre‐existing, but weak, sensitization cannot be ruled out. Examples of allergens reported to cause active sensitization include *p*‐phenylenediamine (PPD) [252, 253], *p*‐tert‐butylcatechol [250, 254], acrylates tested at concentrations historically higher than the present concentrations [255], whereas active sensitization to HEMA 2% pet. has been reported to be extremely rare [256], chloroacetamide [257], Compositae mix [258], Primula extracts [259], isothiazolinones [260], *p*‐tert‐butylphenol formaldehyde resin (PTBP‐FR) [261], dimethyl‐thiocarbamylbenzothiazole sulphide [262], and gold sodium thiosulfate (GST) [263]. Controversy exists over active sensitization to GST due to its characteristic late‐reacting nature [264].

Late patch test reactions should be considered in the differential diagnosis of active sensitization. Such late reactions become positive beyond Day 7 (D7), and are commonly seen with metal salts including gold [74], neomycin and corticosteroids [2].

In general, the risk of sensitization from patch testing with standard allergens is very low, and the benefits far outweigh any potential risks. Therefore, this minor risk should never be a reason to avoid patch testing.

### 10.3 Persisting/Long‐Lasting Reactions

A positive patch test reaction can occasionally persist for several weeks or even months, particularly with allergens such as metal salts (e.g., GST, nickel sulphate, potassium dichromate), acrylates [265] and doxepin [266], among others. Uchida et al. reported a case with a positive patch test reaction to PPD that persisted for > 1 month [267]. Gold salts, particularly GST, are notorious for causing persisting reactions, sometimes lasting for several months [74, 266, 268, 269]. A long‐lasting positive patch test reaction to methyl methacrylate, persisting for up to 12 months, has also been reported [270].

Palladium chloride [271, 272], sodium tetrachloropalladate [273] and GST [274] have been reported to cause persisting granulomatous patch test reactions. Besides, persisting pseudolymphomatous patch test reactions were reported with GST [275, 276], minoxidil [277] and acrylates [278]. The underlying mechanism may involve persistent antigen stimulation and/or impaired down‐regulation of the cell‐mediated immune response. Factors such as a strong initial reaction, age over 60, and atopy have been suggested as risk factors for prolonged patch test reactions [279].

### 10.4 Flare‐Up Reactions

A flare‐up of an existing or healed dermatitis may occur in the course of a (strong) positive patch test reaction. Such flare‐ups usually indicate that one or more of the positively tested allergens is or has been the allergen responsible for the dermatitis [280, 281], linked to a local skin memory response [280].

### 10.5 Pigmentation Changes

A patch test reaction may rarely result in localized transient hyperpigmentation, hypopigmentation or depigmentation (contact leukoderma). Hyperpigmentation has been observed up to 6 months post‐testing, especially in individuals with darker Fitzpatrick skin type IV or skin of colour, making pre‐test counselling for these patients advisable [282].

### 10.6 Immediate Type Reactions

Very rarely, patch testing may cause (usually mild) contact urticaria, occasionally with systemic involvement. According to a recent review summarizing the limited evidence, anaphylaxis or anaphylactoid reactions are extremely rare, estimated to occur in about 1 of 100 000 patients following a survey among US patch test experts [283]. Antibiotics such as bacitracin or neomycin have been described as cause [3, 284]. The immunologic or non‐immunologic nature of these reactions is often unclear. Urticarial dermographism might be triggered from removing the patch test adhesives tapes [3]. Recently, follicular traction urticaria, a novel type of inducible urticaria, was reported to be triggered by patch testing [285]. Immediate type reactions typically disappear within 24 h.

### 10.7 Other Adverse Effects

Contact dermatitis to patch test adhesive tape and skin marker may develop [286, 287]. Other reported adverse effects include the Koebner phenomenon in patients with active psoriasis or lichen planus [284, 288].

### 10.8 Subjective Complaints

Itching, pain or discomfort at patch test sites, particularly during the occlusion period, is commonly observed. This may result from a positive patch test reaction or nonspecific irritation caused by adhesives and prolonged skin occlusion [64]. Some patients feel more itching immediately after removal of the tape [280, 289].

It is recommended that the patient is appropriately informed about serious adverse events, although these are extremely rare, if patch test guidelines are adhered to (95.2%).

## 11 Patient Counselling on Allergen Avoidance

The literature was searched by the use of PubMed with the following (combined) search terms: allergic contact dermatitis, adherence, compliance, contact allergy, contact sensitization, education, information, memory, patch test, recall and counselling. Textbooks were checked manually. Relevant publications were retrieved in March 2025.

### 11.1 Counselling Patients on Allergen Avoidance

ACD may completely resolve following successful counselling of the patient on allergen avoidance, with workplace conditions (employer and accident insurance) being addressed as required, provided that exposure can be sufficiently reduced or eliminated. Following the final reading, all positive patch tests, regardless of relevance (past, present, unknown, cross‐reaction), should be discussed with the patient [64, 290]. Negative patch test results (and differential diagnoses, such as ICD) may also be of interest [291].

Sufficient time should be allowed to provide information in plain language, that is, in a way patients can understand [292]. Counselling starts with naming the allergen, its synonyms, (pseudo) cross‐reactors [142, 290], and related substances (e.g., in case of formaldehyde contact allergy, also the releasers need to be avoided) [64, 293]. Ingredient label reading of cosmetic products and detergents according to the International Nomenclature on Cosmetic Ingredients (INCI) needs to be explained and demonstrated, so that the patients can identify whether a product is free of the allergen; labelling, however, may pose other problems (see below). For medicines, International Non‐proprietary Names (INN) is used, in other fields (e.g., chemical products for occupational or consumer use) Chemical Abstract Service (CAS) numbers and common names. If indicated, it is also advised to inform patients about how to perform a ROAT or use test with products [106, 294]. Generally, care should be taken not to overwhelm the patient with information; visual/written information and follow‐up appointments can help to achieve the best counselling effect.

For each allergen the patient has been diagnosed to be sensitized to, at least the major potential sources of exposure should be listed [294]. Even if no exposure is currently revealed, the patient should be advised how to avoid future skin contact with the allergen. Common exposure sources at home (cosmetics, household detergents) and at the workplace should be considered. Exposure to solid materials (including metal, plastics, rubber, textile, food) or routes (e.g., airborne, by proxy) may also need to be addressed. Some allergens can be present in a wide range of products.

To further enable verification of allergen‐containing sources the use of additional tools may be helpful. For example, nickel‐allergic patients should not only be informed about risk products such as jewellery, but also should be instructed on how to use the nickel spot test on metallic items that are likely to be in prolonged or repetitive (short) contact with their skin [295, 296].

Additionally, some authors recommend to also include information about the mechanism of ACD and, more specifically, to explain the typical time course between allergen exposure and the occurrence of ACD, its resolution after exposure avoidance, and how exposure thresholds are involved. This may prevent unnecessary or extreme avoidance strategies that some patients believe may be required. Furthermore, it may be valuable to explain the function of allergens, so patients can better assess where they are found (or not), thereby supporting better avoidance [290]. The ‘teach‐back method’, that is, asking patients to repeat the most important information to confirm knowledge and clarify misunderstandings, can be useful [290].

Apart from oral information, also the use of written, structured and regularly updated information is equally necessary. Besides a carry‐on allergy pass, also newer strategies, for example, smartphone applications, may be worthwhile to explore. This is of particular importance for patients with positive patch test reactions to fragrances and preservatives [297]. Some authors recommend the use of visual aids to explain different contributing factors such as irritation or constitutional factors [298]. In any case, attention must be given to keep information concise in order to avoid information overload [292]. The recently updated ESCD patient information leaflets (PILs) may serve as a reliable starting point (https://escd.org/patient‐information‐leaflets/, last accessed April 27, 2025).

Of note, written information should not be restricted to just providing the names of the allergens, or allergen handouts [294], but it is important to actually explain the content of these to the patient, and to also point out specific products to avoid and those that are safe to use (e.g., pharmaceuticals, cosmetics) [294, 299], that is, provide ‘black lists’ and ‘white lists’. To help the patient identify safe cosmetic products, databases have been developed [300, 301, 302, 303].

A follow‐up consultation may be scheduled no sooner than 4–8 weeks after the patch test, but probably not later than 3–6 months, also depending on severity of ACD [290, 292], as it is useful to repeat the information at a subsequent consultation. Dedicated programs have been developed, mostly to address hand dermatitis, and also foot eczema, in occupations at risk [304, 305].

- It is recommended to discuss all positive patch test results and explain allergen names, synonyms, cross‐reactors and typical exposure sources (100%).
- It is recommended to demonstrate reading of labels of cosmetics products (INCI),^a^ detergents (INCI),^a^ medicines (INN),^b^ and chemical products (CAS),^c^ and explain ROAT^d^ and use testing, where indicated (100%).
- It is recommended to provide concise written and oral information, for example, using the ‘teach‐back’ method (95.2%).
- It is suggested to support patients with additional tools like an allergy card, spot tests and safe product databases (100%).
- It is recommended to provide information on skin protection, including correct glove use (100%).
- It is recommended to schedule follow‐up consultations to repeat or review information, re‐assess the skin condition and clinical relevance of positive patch test reactions, discuss possible treatments, evaluate quality of life and recommend work‐related adjustments as needed (100%).

^a^INCI: International Nomenclature of Cosmetic Ingredients; ^b^INN, International non‐proprietary Names; ^c^CAS, Chemical Abstract Service; ^d^ROAT, repeated open application test.

### 11.2 Challenges in Patient Counselling

Individuals from (educationally) disadvantaged backgrounds and with reduced personal resources may find it more difficult to capture all information; also, reading and understanding of ingredient labels on cosmetic products may be difficult to anyone [297]. Patients also need to remember their allergens (‘allergen awareness’), yet recall is often only moderate and may differ according to sex, age, memory, educational and socioeconomic level, time since patch testing, the number of allergens found, type and complexity of the allergen name, the occupational context (if present), atopy and severity and prolonged duration of the skin disease [306, 307]; moreover, compliance may differ between patients. A UK study showed that, among 135 patch tested patients, around 25% could not even recall having received any information about their test results 2–3 months later [308]. A US survey, including 757 patch tested patients who were given a questionnaire on average 13 months after patch testing (the mean age of the patients was 59 years), showed that only 50% of 238 patients with positive patch test reactions to one or two allergens remembered their allergies [309]. Moreover, among 342 patients with three or more positive patch test reactions, only 24% remembered all their allergies correctly.

Notwithstanding conflicting results, there appears to be a tendency for better recall among women, younger patients and individuals with a higher level of education, those who underwent patch testing more recently, if the number of allergens found was lower and when it concerned less complex substances, less severe skin disease of a shorter duration [306, 307, 308, 309, 310]. This indicates that populations should be identified that may benefit from more careful information, a topic that requires more research [307]. Moreover, allergen groups (e.g., fragrances) appear to be remembered better than individual allergens [306]. Some authors therefore recommend grouping allergens for counselling (e.g., fragrances, preservatives, dyes) [292] and including this in the explanations given to patients. For a summary of possible interventions to improve recall and avoidance strategies, see [306]. Apart from information developed by dermato‐allergology societies (ESCD, ACDS), numerous websites and online support communities on various social media platforms exist, but the quality varies considerably, depending on whether the information is provided by physicians, fellow patients, industry or ‘skinfluencers’ [311, 312, 313].

Even if patients are well aware of their allergens, avoidance may still be difficult [64], and resolution of ACD may remain a challenge. Examples include:

- Allergens may be ubiquitous [314], hidden (e.g., mixtures‐in‐mixtures or formaldehyde) [165], undeclared [170], unlabelled or mislabelled [315] or patients are exposed indirectly, such as via ingestion or the airborne route (e.g., fragrances, sesquiterpene lactones, preservatives).
- Marketing terms such as ‘hypoallergenic’ can be misleading and not used for guidance.
- Natural substances (e.g., *Myroxylon pereirae* resin) are often considered innocuous by many consumers and this should be clearly debunked in those sensitized [316, 317].
- Cross‐reactors, which may not always be fully known, may still trigger relapses or aggravation of dermatitis.
- A socially driven desire to continue using a certain product can affect adherence; for example, hair dyeing or use of nail acrylates [318]. Employers may be unwilling or unable to sufficiently modify exposure conditions.
- In case of multifactorial disease (e.g., hand dermatitis), it is important to explain, besides allergies, all other potentially contributing factors, such as irritant exposures (e.g., wet work) and constitutional factors (e.g., atopy). Some degree of chronicity of the skin condition cannot be excluded despite these efforts, and additional therapeutic actions may be needed.

In case allergen avoidance seems to have no positive impact whatsoever, re‐assessment may be necessary, for example, verifying if avoidance is effectively achieved, or if overlooked or unusual allergen sources may still play a role. Supplementary testing to uncover additional allergens may need to be considered. It should be noted that longstanding and/or severe hand dermatitis and occupational dermatoses may have a poor prognosis, even after avoidance of all allergens to which sensitization has been diagnosed. ‘Persistent post‐occupational dermatitis’ could be considered in some patients in whom allergens may have caused such profound damage that chronic eczema persists even if the allergen is completely avoided, for example, in chromium allergy [319].

- It may be considered to recommend smartphone apps and other aids to support allergen identification (95.2%).
- It is suggested to inform patients about hidden, undeclared or mislabelled allergens and to debunk myths around ‘natural’ or ‘hypoallergenic’ claims (95.2%).
- It is recommended to emphasize the role of other factors in chronic dermatitis such as irritants or atopy, if present, and to reinforce skincare and protection measures (100%).
- It is recommended to consider, if avoidance fails, for example, overlooked exposures, additional allergens or alternative diagnoses upon follow‐up (100%).

## 12 Professional Training in Cutaneous Allergy

In this section, the term ‘cutaneous allergy’ is used to comprise contact allergy (delayed‐type hypersensitivity) and contact urticaria/protein contact dermatitis (immediate‐type/combined‐type hypersensitivity). Investigation of cutaneous allergy is time‐intensive, usually requiring a minimum of three visits over 5–7 days and, for effective use of resources, it is important for the patient to be seen at an appropriate centre from the outset. Specialty training in dermatology provides core skills to develop the specific competencies required to practice independently as a dermatologist or allergist. The European Training Requirements (ETR) of the European Union of Medical Specialists (UEMS) give an overview over the knowledge, skills, and competencies that a specialist must acquire during their training in order to practice independently and safely in their chosen specialty [320, 321, 322, 323]. We consider this background of training to be the minimum to enable an individual to fully consider the differential diagnosis and management of a patient with a potential cutaneous allergic reaction. Aspects of immediate‐type hypersensitivity skin testing (prick test) are not considered here.

### 12.1 Physicians With an Interest in Cutaneous Allergy

For physicians who spend a major part of their working career in the field of cutaneous allergy, a higher level of training should be expected [324]. Specialist centres may provide diagnostic services for complex cases, for example those involving outbreaks of ACD in the workplace [216, 325] or wider community, multiple sensitizations and photo‐allergy. Factory or workplace visits, specialist patch and photo testing and specialist pharmacy services to dilute patient samples are sometimes needed. An individual would be expected to gain the knowledge and skills in cutaneous allergy set out below (beyond the dermatology and allergy core skills) during an indicative duration of training of 12 months, with 250–300 patients being seen during this period to achieve competence. The skills related to this field of work are indicatively described in a document by the British Association of Dermatologists [324].

### 12.2 Maintenance of Expertise

Minimum standards for provision of a cutaneous allergy service have been defined, for example, in the United Kingdom [326]. To maintain competence, it was recommended that clinicians should investigate at least 100 cases a year [326]. Investigation of cutaneous allergy is delivered by a multiprofessional team. The team should have regular meetings (at least four times a year). The broad aim of these regular clinical governance meetings is to ensure that the service is focused on the need to provide timely, safe and effective services to patients, for example, reviewing activities since the previous meeting, waiting list data to assess demands on the service and issues regarding service delivery, adverse events and discussing difficult or instructive cases.

It is recommended that results from investigations should be recorded in a database with a minimum dataset and critically compared with national pooled results as part of departmental governance procedures; see also Section 13.

There is a need for ongoing training of team members. To ensure a uniform inter‐individual patch test reading technique, continuous training is necessary, for example in the context of ‘patch test courses’ at scientific meetings. As another possibility, an online patch test reading course is provided by the German contact dermatitis research group (https://ablesetraining.ivdk.org/, last accessed 2 February 2026; registration with DocCheck needed). New evidence‐based practice, research, national standards, guidance and audit results all need to be disseminated to staff, to ensure the implementation of procedures that achieve quality outcomes. Training and Clinical Professional Development should be discussed and planned to ensure that all team members fulfil professional requirements to be fully up to date [326]. It is recommended that the team lead attend update meetings on contact allergy at least once every year. The unit should have up‐to‐date reference books on contact allergy, including occupational skin disease and access to relevant journals.

## 13 Databases and Surveillance

In the practice of patch testing, from the perspective of the attending physician and researcher, respectively, the term ‘databases’ refers to two aspects: (i) retrieval of information for patient management or scientific publication, and (ii) collection of departmental patch test results, usually with a view to later analysis and publication. Sufficiently standardized patch test data collected in the course of several years and/or by different centres can serve the important purpose of contact allergy surveillance, that is, the observation of time trends or geographical differences in sensitization prevalences, for example, pinpointing differences between Europe and North America with regard to containing, or not, the methylisothiazolinone contact allergy epidemic [327]. In this section, key issues of both aspects are briefly outlined; for further details, see [328].

### 13.1 Information Sources

The internet offers a wealth of accessible information; however, validity and completeness of content varies. This may be an issue particularly for patients seeking information prior to, or perhaps even instead of, a specialist consultation (which may not be easily available) [329]. Regarding product information, the full INCI labelling information of cosmetics can often be found on the manufacturer's website if the patient is unable to produce the package. However, the amount and accessibility of information offered by different manufacturers vary greatly. In case of medicinal products (both topical and systemic), freely available Summaries of Product Characteristics (SmPC) are provided by regulatory bodies, either national and international (e.g., European Medicines Agency, EMA, https://www.ema.europa.eu/en/medicines, last accessed 8 May 2025). In‐depth information on pharmacological substances can be found in official pharmacopoeias, which typically are available to paying subscribers (e.g., European Pharmacopoeia Online, https://pheur.edqm.eu/home, last accessed 8 May 2025). In other cases such as chemical products and substances for professional or consumer use, or medical devices, it is helpful to download SDSs provided by manufacturers (obligatory in the EU and other countries); however, they often provide limited information and may not always be correct [151, 166]. Chemical and toxicological information on haptens is available from services that either need subscription (such as the Chemical Abstract Service, CAS) or freely, e.g., PubChem (https://pubchem.ncbi.nlm.nih.gov/, last accessed 24 July 2025). The following list includes some further selected resources in the English language:

- CosIng database (https://ec.europa.eu/growth/tools‐databases/cosing/, last accessed 8 May 2025): List of all cosmetic ingredients, including INCI names, functions and (links to) regulatory information.
- Expert opinions of the Scientific Committee on Consumer Safety and its predecessors on the safety of cosmetic ingredients (https://health.ec.europa.eu/scientific‐committees/scientific‐committee‐consumer‐safety‐sccs/sccs‐opinions_en, last accessed 8 May 2025). Older opinions may be found following information found in CosIng.
- Toxicological monographs, including on contact sensitization, by the German MAK Commission (https://series.publisso.de/en/pgseries/overview/mak, last accessed 8 May 2025); only partly available in English and Spanish.

### 13.2 Software for Documentation of Patch Test Results

There are several options regarding software suitable for the documentation of patch test results, along with relevant demographic and clinical data. These have been briefly reviewed [328].

### 13.3 Contact Allergy Networks and Surveillance

A collection of results of all patch tested patients, that is, (also including completely negative cases for representativeness), by one department already offers interesting possibilities for data analysis. However, these possibilities are vastly increased by joining a (national) data network, including, but not limited to, benchmarking and quality control of one department's results against the average of the peer group, with the possibility of enhancing standardization and quality [68]. The benefit of contact allergy surveillance has been proven multiple times [328]. Beyond evident use for monitoring contact allergy prevalences for common (baseline series) or occupational allergens, the sheer size of such data collections also enables to focus on special, small subgroups [330] or (very) rare allergens [331].

### 13.4 Cosmetovigilance/Pharmacovigilance

In the context presented here, cosmetovigilance (or, similarly, pharmacovigilance) describes different concepts of a special type of contact allergy surveillance implemented by dermatologists or other allergy specialists, depending on the country's context. It is mainly based on case reports with a full work‐up in terms of cosmetic product ingredient testing, possibly within a network of dermatologists [328]. An early example is the French REVIDAL/GERDA system [332]. The outcome of such dermatological vigilance cannot be underestimated in terms of informing on new, possibly emerging, contact allergens or new sources of exposure to known sensitizers.

It is recommended to collect, archive and share patch test data, including negative test results and patients' basic demographic and clinical information, as this is invaluable for (i) auditing of departmental results to assure best diagnostic practices on a local and network level, and (ii) scientific analyses to provide efficient epidemiological surveillance of contact allergies (100%).

## Author Contributions

**Tove Agner:** writing – original draft, writing – review and editing. **Margarida Gonçalo:** writing – original draft, writing – review and editing. **Marie‐Noёlle Crépy:** writing – original draft, writing – review and editing. **Richard Brans:** writing – original draft, writing – review and editing. **Olivier Aerts:** writing – original draft, writing – review and editing. **Magnus Bruze:** writing – original draft, writing – review and editing. **Carola Lidén:** writing – original draft, writing – review and editing. **Alicia Cannavó:** writing – original draft, writing – review and editing. **Ana M. Giménez‐Arnau:** writing – original draft, writing – review and editing. **Maria Pesonen:** writing – original draft, writing – review and editing. **Wolfgang Uter:** writing – original draft, writing – review and editing, conceptualization, methodology, software. **Mihaly Matura:** writing – original draft, writing – review and editing. **An Goossens:** writing – original draft, writing – review and editing. **Radoslaw Spiewak:** writing – original draft, writing – review and editing. **Vera Mahler:** writing – original draft, writing – review and editing. **Thomas Rustemeyer:** writing – review and editing, writing – original draft. **Swen M. John:** writing – original draft, writing – review and editing. **Jeanne Duus Johansen:** writing – original draft, writing – review and editing, conceptualization, methodology, supervision. **Esen Özkaya:** writing – original draft, writing – review and editing. **S. Mark Wilkinson:** writing – original draft, writing – review and editing. **Suzana Ljubojević Hadžavdić:** writing – review and editing, writing – original draft. **Luca Stingeni:** writing – original draft, writing – review and editing. **Ian R. White:** writing – original draft, writing – review and editing. **Nadia Raison‐Peyron:** writing – original draft, writing – review and editing.

## Disclaimer

As regards V. Mahler, the positions expressed in this guideline are the personal views of the author and may not be understood or quoted as being made on behalf of or reflecting the position of the respective national competent authority, the European Medicines Agency, or one of its committees or working parties.

## Conflicts of Interest

Olivier Aerts is investigator, speaker and/or consultant for Leo Pharma, Abbvie, Sanofi‐Genzyme, L'Oréal/La Roche Posay, Bioderma/NAOS, Novartis, Amgen. Tove Agner has been a speaker/consultant/advisor for AbbVie, Almirall, Eli Lilly, LEO Pharma, Pfizer and Sanofi‐Genzyme. Richard Brans has been speaker/consultant for LEO Pharma. Marie‐Noёlle Crépy is investigator, speaker and/or consultant for LEO Pharma, Sanofi‐Genzyme, AbbVie and Pfizer. Ana M. Giménez‐Arnau, is or recently was a speaker and/or advisor for and/or has received research funding from Almirall, Amgen, AstraZeneca, Avene, Blue ‐Print, Celltrion, Celldex, Escient Pharmaceutials, Genentech, GSK, Harmonic Bio, Incyte, Instituto Carlos III‐ FEDER, Jaspers, Leo Pharma, Menarini, Mitsubishi Tanabe Pharma, Noucor, Novartis, Sanofi**–**Regeneron, Septerna, Servier, Thermo Fisher Scientific, Uriach Pharma. Margarida Gonçalo has been advisor and or lecturer or participated in clinical trials from Abbvie, Almirall, Astra‐Zeneca, Amgen, Biogen, Byocrist, Lilly, LEO Pharma, Johnson, Novartis, Pfizer, Sanofi, Takeda. Swen M. John has received lecture fees from Sanofi‐Genzyme and LEO Pharma. Suzana Ljubojević Hadžavdić is investigator for atopic dermatitis in a clinical study (Abbvie, Amgen, Nektar) and for a study on chronic spontaneous urticaria (Novartis); lecturer for Novartis, Abbvie, Sanofi, Pliva, Bayer and Berlin‐Chemie. Esen Özkaya is investigator, speaker and/or consultant for Amgen, Novartis, Sanofi Genzyme, Pfizer. Maria Pesonen has received lecture fees from UCB Pharma, Finland. Nadia Raison‐Peyron received fees for educational lectures from ALK Abello. Radoslaw Spiewak is part‐time employee and shareholder at the Instytut Dermatologii, Krakow, Poland; speaker/writer for Polish Society of Allergology, Polish Dermatological Society, Medycyna Praktyczna, Forum Media Polska, MedicaExpert, Chiesi, Leo Pharma. Luca Stingeni is investigator and speaker for AbbVie, Almirall, Amgen, BMS, Eli Lilly, L'Oreal, LEO Pharma, Novartis, Pfizer, Sanofi‐Genzyme, UCB. Wolfgang Uter has accepted research funds directed to the department from the IDEA project (IFRA, https://ifrafragrance.org/). The other authors declare no conflicts of interest.

## Data Availability

The data that support the findings of this study are available in PubMed/Medline at https://pubmed.ncbi.nlm.nih.gov. These data were derived from the following resources available in the public domain: ‐ PubMed/Medline, https://pubmed.ncbi.nlm.nih.gov.
